# Life-course socioeconomic conditions, multimorbidity and polypharmacy in older adults: A retrospective cohort study

**DOI:** 10.1371/journal.pone.0271298

**Published:** 2022-08-02

**Authors:** Katharina Tabea Jungo, Boris Cheval, Stefan Sieber, Bernadette Wilhelmina Antonia van der Linden, Andreas Ihle, Cristian Carmeli, Arnaud Chiolero, Sven Streit, Stéphane Cullati

**Affiliations:** 1 Institute of Primary Health Care (BIHAM), University of Bern, Bern, Switzerland; 2 Swiss Center for Affective Sciences, University of Geneva, Geneva, Switzerland; 3 Laboratory for the Study of Emotion Elicitation and Expression (E3Lab), University of Geneva, Geneva, Switzerland; 4 Swiss NCCR “LIVES: Overcoming Vulnerability: Life Course Perspectives”, University of Geneva, Geneva, Switzerland; 5 LIVES Centre, Swiss Centre of Expertise in Life Course Research, University of Lausanne, Lausanne, Switzerland; 6 Center for the Interdisciplinary Study of Gerontology and Vulnerability, University of Geneva, Geneva, Switzerland; 7 Population Aging Research Center and Population Studies Center, University of Pennsylvania, Philadelphia, PA, United States of America; 8 Population Health Laboratory, #PopHealthLab, University of Fribourg, Fribourg, Switzerland; 9 Department of Psychology, University of Geneva, Geneva, Switzerland; 10 School of Global and Population Health, McGill University, Montreal, Canada; 11 Department of Readaptation and Geriatrics, University of Geneva, Geneva, Switzerland; South African Medical Research Council, SOUTH AFRICA

## Abstract

Socioeconomic conditions across the life course may contribute to differences in multimorbidity and polypharmacy in old age. However, whether the risk of multimorbidity changes during ageing and whether life-course socioeconomic conditions are associated with polypharmacy remain unclear. We investigated whether disadvantaged childhood socioeconomic conditions (CSCs) predict increased odds of multimorbidity and polypharmacy in older adults, whether CSCs remain associated when adjusting for adulthood socioeconomic conditions (ACSs), and whether CSCs and ACSs are associated cumulatively over the life course. We used data for 31,432 participants (multimorbidity cohort, mean [SD] age 66·2[9] years), and 21,794 participants (polypharmacy cohort, mean age 69·0[8.9] years) from the Survey of Health, Ageing, and Retirement in Europe (age range 50–96 years). We used mixed-effects logistic regression to assess the associations of CSCs, ASCs, and a life-course socioeconomic conditions score (0–8; 8, most advantaged) with multimorbidity (≥2 chronic conditions) and polypharmacy (≥5 drugs taken daily). We found an association between CSCs and multimorbidity (reference: most disadvantaged; disadvantaged: odds ratio (OR) = 0·79, 95% confidence interval (CI) 0·70–0·90; middle: OR = 0·60; 95%CI 0·53–0·68; advantaged: OR = 0·52, 95%CI 0·45–0·60, most advantaged: OR = 0·40, 95%CI 0·34–0·48) but not polypharmacy. This multimorbidity association was attenuated but remained significant after adjusting for ASCs. The life-course socioeconomic conditions score was associated with multimorbidity and polypharmacy. We did not find an association between CSCs, life-course socioeconomic conditions, and change in odds of multimorbidity and polypharmacy with ageing. Exposure to disadvantaged socioeconomic conditions in childhood or over the entire life-course could predict multimorbidity in older age.

## Introduction

Multimorbidity, defined as the co-existence of multiple chronic conditions [1], is highly prevalent in older adults. More than 50% of adults aged ≥50 years have ≥2 chronic conditions, and prevalence rates increase with age [2, 3]. Multimorbidity can lead to cognitive decline, functional decline, and loss of quality of life. Treatment of multimorbidity is challenging for healthcare systems [4] and will increase in the future because the prevalence of multimorbidity is predicted to increase with population ageing [5]. Multimorbidity is often accompanied by polypharmacy, defined as the concurrent intake of multiple medications [6]. Appropriate polypharmacy improves health outcomes, but inappropriate polypharmacy may have detrimental health effects, such as adverse drug events and cognitive decline [7].

The risk of chronic conditions in older adults is linked to life-course socioeconomic conditions (SECs) at different life stages [8–11]. For example, childhood SEC (CSC) were found to be associated with different indicators of older adults’ health and wellbeing such as quality of life, lung function, grip strength, and depressive symptoms, with more advantaged childhood conditions leading to better outcomes [12–15]. Another study found that adjusting for CSC “consistently attenuated the magnitude of the educational gradient”, but that there are country differences in the “degree to which childhood health and [socioeconomic position] impact subsequent educational gradients in health” [16]. A recent systematic review, however, found that the findings related to the association between childhood household determinants (e.g., paternal social class, etc.) and multimorbidity was mixed [17].

Different mechanisms, which are not mutually exclusive, may underlie the association between CSCs and health outcomes in older adults [18–20]. In this study, 2 main mechanisms were hypothesized: the accumulation of exposures to poor socioeconomic circumstances and conditions for multimorbidity/polypharmacy throughout the life course versus childhood being a critical or sensitive period in the life course for multimorbidity/polypharmacy in older age. Accumulation of exposures describes a succession of exposures to socio-economic living conditions that are unfavourable to health at different points of the life course. This accumulation of exposures increases the risk of poor health in old age. The critical or sensitive period model focuses on the timing of exposures. It states that during the developmental phase of the individual, being exposed to a risk factor could result in irreversible damage to the body (critical period) or reversible damage after a certain time (sensitive period), whereas the same exposure to the risk factor outside the critical or sensitive period would not have the same impact on the body. Three large population-based studies found an association between CSCs and the mean number of chronic conditions in older adults [10, 11, 21]. However, in these studies, this association was largely explained by adulthood SECs (ASCs). Pavela and Latham argued that the overall impact of CSCs works primarily through ACSs, which agrees with the accumulation of risk model [10]. These findings contrast with the critical period hypothesis and point to an accumulation of exposures to poor SECs throughout the life course. However, no previous study used a life-course score to test whether CSCs and ACSs are accumulative over the life course. Finally, to the best of our knowledge, no study has examined life-course SEC associations with polypharmacy.

Because of the complex nature of the ageing process, we need to shift our focus from single diseases and specific periods to longitudinal, life-course health trajectories to gain a better understanding of healthy ageing and its determinants [22]. Despite previous research on the association between life-course conditions and multimorbidity in older people and an increased risk as cross-sectional age increases [10, 23–25], there has been limited research on the association between different CSC categories and the change in odds of multimorbidity/polypharmacy during the ageing process within individuals. We embrace the cumulative advantage/disadvantage hypothesis [23], which argues that exposure as well as the accumulation of exposures generate growing differences in old age. However, we also embrace the idea in the Strachan & Sheik model, which stipulates that CSCs affect the level of health and the speed of health decline [20]. Thus, here, we also examined the association between CSCs and the odds of multimorbidity during ageing. Based on these hypotheses, we performed the same type of analyses for polypharmacy.

We had 3 study objectives: to assess 1) whether disadvantaged CSCs predict increased odds of multimorbidity and polypharmacy in older adults and whether disadvantaged CSCs change the odds of multimorbidity/polypharmacy during ageing, 2) whether CSCs remain associated when adjusting for ACSs, and 3) whether CSCs and ACSs are associated cumulatively over the life course.

## Methods

### Data source

We used longitudinal and multinational data from the Survey of Health, Ageing and Retirement in Europe (SHARE) [24], in which data have been collected in 7 waves, every 2 years, since 2004 in adults aged ≥50 years. Information on chronic conditions was assessed in all waves except for wave 3. Polypharmacy was assessed in waves 6 and 7. Waves 3 and 7 measured retrospective life-course data including early-life SECs and main occupational position during adult life. In this study, we included participants who participated in wave 3 or 7 and had at least one assessment of multiple chronic conditions or polypharmacy. Two figures in the supporting materials provide flow charts of participant inclusion (S1 and S2 Figs in S1 File).

### Main independent predictors

#### Childhood SECs (CSCs)

We computed CSC based on the Wahrendorf et al. measure of childhood circumstances, which combines 4 binary indicators of SECs at age 10, namely occupational position of the main breadwinner, number of books in the household, overcrowding, and housing quality [25]. Each indicator is coded 0 for “advantaged” and 1 for “disadvantaged” and combined in a score ranging from 0 (“most advantaged”) to 4 (“most disadvantaged”). These 4 indicators have previously been found relevant for assessing the long-term effect of CSCs on health outcomes [26–29]. The SHARELIFE module of the SHARE study, a retrospective survey of participants’ life histories, collected this information [30]. We reconstructed the occupational position of the main breadwinner based on the 10 main occupational groups of the International Standard Classification of Occupations according to skill levels, as previously done [25]. We classified the first and second skill levels as “low”, and the third and fourth levels as “high” [31]. We constructed a binary variable for the number of books in the household. In line with previous research, we assumed the category “0–10 books” as an indicator of social disadvantage [28]. The variable “overcrowding” was calculated based on the number of rooms (kitchen, bathrooms, and hallways excluded) and the number of people living in the household. We considered the apartment/house to be overcrowded when there was more than one person per room [29]. Lastly, we assessed the housing quality based on a fixed bath, cold running water supply, hot running water supply, indoor toilet, and central heating. In line with previous research, we considered the household as disadvantageous if none of these factors was present [27].

#### Adulthood SECs (ASCs)

We used the following indicators to measure ACSs: highest educational, occupational class, and financial strain. The educational attainment was classified as 0, primary; 1, secondary; and 2, tertiary. The occupational class was defined using the International Standard Classification of Occupations classification of the main job that the participant held during their working life: 2, high skilled; 1, low skilled; and 0, never worked. Financial strain was assessed with the question “Thinking of your household’s total monthly income, would you say that your household is able to make ends meet?” The answer options were with great difficulty (weight 0; with difficulty, 0; fairly easily, 1; and easily, 2).

#### Life-course SECs score

To assess whether CSCs and ACSs combined predict our outcomes (objective 3), we used a life-course SECs score (hereafter life-course score) that combines CSCs and ACSs and has been used in previous research [32]. In this score, each life-course period has the weight of 2 (meaning that each life-course period–childhood, young adulthood, middle age, and old age—received the same weight). For CSC in this score, we created a categorical variable by aggregating the information of the above-mentioned 4 indicators (occupational position of the main breadwinner, number of books in the household, overcrowding, and housing quality). For each indicator, 0 corresponds to a disadvantaged situation and 0.5 to an advantaged situation. For young adulthood, we used the educational status. For middle age, we used the main occupational class. For old age, we used the variable measuring financial strain. These variables were coded as defined in the paragraph above. In total, this led to a score that ranged from 0 to 8 with 0 indicating a disadvantaged life-course or a maximal exposure to disadvantaged SECs during the whole life-course and 8 an advantaged life-course or no exposure to disadvantaged SECs during the whole life-course.

### Outcomes

Chronic conditions were assessed by this question: “Has a doctor ever told you that you had / Do you currently have any of the conditions on this card?”. The card that included a list of the conditions: heart attack including myocardial infarction or coronary thrombosis or any other heart problem including congestive heart failure; high blood pressure or hypertension; high blood cholesterol; stroke or cerebral vascular disease; diabetes or high blood sugar; chronic lung disease such as chronic bronchitis or emphysema; cancer or malignant and benign tumour, including leukaemia or lymphoma but excluding minor skin cancers; stomach or duodenal ulcer, peptic ulcer; Parkinson’s disease; cataracts; hip fracture; other fractures; Alzheimer’s disease, dementia, organic brain syndrome, senility or any other serious memory impairment; other affective or emotional disorders, including anxiety, nervous or psychiatric problems; rheumatoid arthritis; and osteoarthritis, or other rheumatism. Participants specified that a doctor had told them that they had this condition and that they were currently being treated for or bothered by this condition. Except for cancer, the date of the diagnosis was not available. In this study, we worked with a definition that stated that chronic conditions should last minimum three months [33]. Consequently, we excluded cataracts, hip fractures, and other factures because of their commonly acute nature. All conditions were collected at each wave, except for rheumatoid arthritis, osteoarthritis, and rheumatism, which were collected in waves 1, 2 and 4 only, and benign tumours in waves 2 and 4 only. We defined multimorbidity as the coexistence of ≥2 chronic conditions [1]. Polypharmacy was assessed by asking participants, “Do you take at least five drugs on a typical day?”, which is the most frequently used threshold [6]. This question captures drugs prescribed by physicians and also over-the-counter medications and dietary supplements, such as vitamins and minerals.

### Covariates

For both analyses, the covariates were linear age, squared age, sex, birth cohort (1919–1928, 1929–1938, 1939–1945, and post-1945), and country (Belgium, Austria, Croatia, Czech Republic, Denmark, Estonia, France, Germany, Greece, Israel, Italy, Luxembourg, Poland, Slovenia, Spain, Sweden, Switzerland). The covariates for the multimorbidity analyses were known lifestyle risk factors: obesity (≥30 kg/m2), alcohol consumption, smoking, and physical activity. All these risk factors were self-reported. Two variables were used to assess the level of daily life physical activity [34]. The first item assessed vigorous physical activity (“How often do you engage in vigorous physical activity, such as sports, heavy housework, or a job that involves physical labour?”). The second item assessed moderate physical activity (“How often do you engage in activities that require a low or moderate level of energy such as gardening, cleaning the car, or doing a walk?”). Participants answered by using a 4-point scale (1, >1 a week; 2, once a week; 3, 1–3 times a month; 4, hardly ever, or never). Participants who did not answer “1” to either question were classified as “physically inactive”. For the polypharmacy analyses, the models were also adjusted by the number of chronic conditions, depression (≥4 on EURO-Depression scale), limitations in activities of daily living (≥1 on activities of daily living scale) [35] and living situation. In multimorbidity models, participants’ follow-up status (dropout, deceased, no dropout) was included in the models to adjust for attrition. Assessing dropout was not possible for polypharmacy because it was assessed only during the last 2 waves.

### Data analysis

Multimorbidity and polypharmacy data were estimated by using logistic mixed-effect models, which account for the nested structure of the data (e.g., multiple observations within participants). All models included random intercepts for participants. To identify the best random structure, we tested nested models with various random effects and assessed them on the basis of the Bayesian information criterion as well as likelihood ratio tests. A random structure with slopes (based on age) was tested but not included because it did not improve the models. For time-varying variables (financial strain, obesity, physical activity, cognitive performance, multimorbidity and as additional covariates in polypharmacy models depression, limitations in activities of daily living, and living situation), we introduced a time lag to limit reverse causation bias. Specifically, for a given wave (except wave 1), we assigned the value of the preceding wave for each of these variables.

Model 1 assessed the associations between CSCs and multimorbidity (Table 2) and polypharmacy (Table 4) in older adults. We centred age at 73 years and divided by 10, so the coefficients must be interpreted as the overall change over a 10-year interval. A visual examination of the outcomes showed an increase with age, so we added squared age in the models to account for the quadratic pattern of odds of multimorbidity and polypharmacy over age. Model 2 adjusted for indicators of ACSs (education, main occupational position, and financial strain). Model 3 additionally included the above-mentioned multimorbidity/polypharmacy risk factors.

To investigate whether CSCs moderated an increase in odds of multimorbidity/polypharmacy during ageing, we added an interaction term between CSCs and age to Model 1, 2, and 3. A significant interaction is interpreted as a different rate of increase of the outcomes in different CSC sub-groups (S3 and S5 Tables in S1 File).

We also assessed the association between the life-course score with multimorbidity (Table 3) and polypharmacy (Table 5) at age 73 years (model 1). Models 2 additionally adjusted for multimorbidity/polypharmacy risk factors.

In addition, we assessed the association between the life-course score and the rate of change in odds of multimorbidity and polypharmacy as participants become older (S4 and S6 Tables in S1 File). Models 1b and 2b additionally included interaction terms of the life-course score with (linear and squared) age and multimorbidity/polypharmacy risk factors. In models 2a and 2b, the interactions between the covariates and age (linear and quadratic) were added to properly control for these covariates on the development of odds of multimorbidity and polypharmacy across age.

The main analyses were replicated with sex stratification (S7 to S10 Tables in S1 File) to explore sex differences, as previous studies have found sex differences in multimorbidity and polypharmacy trends [36]. The baseline table including the number of participants per country and the main results including country coefficients are presented in the S11–S15 Tables in S1 File. P<0.05 was considered statistically significant.

Four sensitivity analyses were performed (see S16 Table in S1 File). Three examined issues related to participants’ attrition: first, excluding participants who died during follow-up; second, excluding participants who dropped out during follow-up; third, excluding participants aged ≥ 90 years old. A fourth analysis adjusted the models with 2 indicators of cognitive performance (memory and verbal fluency) because cognition is associated with multimorbidity [37, 38]. Adjustment on cognition was not included in the main results because of the decrease in respondents.

Three robustness analyses were performed (see S17 Table in S1 File). First, we replicated the analyses by treating multimorbidity as a continuous score, using linear mixed regression models. Second, we included the category “other” in the score of chronic conditions. Third, we imputed the last recorded value to the following waves for asthma, arthritis, osteoporosis (measured in waves 1, 2 and 4) and benign tumours (measured in waves 2 and 4). All sensitivity and robustness analyses repeated the analytical plan of the main results.

### Ethical approval

SHARE was approved by the competent research ethics committees in the participating countries, and all participants provided written informed consent.

## Results

Participant characteristics are reported in Table 1. The sample for the multimorbidity analyses was 31,432 (50·5% women) and for the polypharmacy analyses was 21,794 (50·8% women). The mean (SD) age was 66·2 (9·0) years in the multimorbidity cohort and 69·0 (8·9) years in the polypharmacy cohort. For both cohorts, the age range was 50–96 years.

**Table 1:** Baseline characteristics of study participants.

		Cohort for multimorbidity analyses	Cohort for polypharmacy analyses
		(N = 31,432)	(N = 21,794)
*Age*, *mean (SD)*		66·2 (9·0)	69·0 (8·9)
*Attrition*			
	No dropout	27,163 (86·4)	21,527 (98·8)
	Dropout	3,059 (9·7)	0 (0)
	Deceased	1,210 (3·9)	267 (1·2)
*Sex*			
	Women	15,910 (50·6)	11,075 (50·8)
	Men	15,522 (49·4)	10,719 (49·2)
*Birth cohort*, *n (%)*			
	1919–1928	1,331 (4·2)	701 (3·2)
	1929–1938	5,415 (17·3)	3,978 (18·3)
	1939–1945	6,923 (22·0)	5,134 (23·6)
	After 1945	17,763 (56·5)	11,981 (55·0)
*Polypharmacy*, *n (%)*		-	5,318 (24·4)
*Multimorbidity*^*3*^, *n (%)*		11,456 (36·5)	9,616 (44·1)
*Childhood socioeconomic conditions*, *n (%)*			
	Most disadvantaged	4,061 (12·9)	3,017 (13·8)
	Disadvantaged	7,203 (22·9)	5,163 (23·7)
	Middle	10,786 (34·3)	7,348 (33·7)
	Advantaged	7,028 (22·4)	4,732 (21·7)
	Most advantaged	2,354 (7·5)	1,534 (7·0)
*Education*, *n (%)*			
	Primary	5,941 (18·9)	4,320 (19·8)
	Secondary	18,011 (57·3)	12,398 (56·9)
	Tertiary	7,480 (23·8)	5,076 (23·3)
*Main occupational position*, *n (%)*			
	Low skilled	10,917 (63·4)	13,738 (63·0)
	High skilled	10,244 (32·6)	7,133 (32·7)
	Never worked	1,271 (4·0)	923 (4·2)
*Financial strain*, *n (%) (able to make ends meet)*			
	Easily	10,182 (32·4)	8,038 (36·9)
	Fairly easily	10,386 (33·0)	6,226 (28·6)
	With some difficulty	2,929 (9·3)	2,059 (9·5)
	With great difficulty	7,935 (25·3)	5,471 (25·1)
*Obesity*^*1*^, *n (%)*		6,152 (19·6)	5,121 (23·5)
*Alcohol consumption*^*2*^, *n (%)*			
	Ok	23,758 (75·6)	16,607 (76·2)
	Too much	7,674 (24·4)	5,187 (23·8)
*Smoking*, *n (%)*		7,770 (24·7)	5,235 (24·0)
*High physical activity (%)* ^*6*^		6,960 (22·1)	5,596 (25·7)
*Limitation in activities of daily living*^*4*^, *n (%)*		-	1,880 (8·6)
*Nursing home*, *n (%)*			
	*Permanently or temporarily*	-	30 (0·1)
	*Not living in nursing home*	-	21,764 (99·9)
*Depression*^*5*^, *n (%)*		-	5,349 (24·5)

^1^ BMI ≥30 kg/m^2^

^2^ self-reported

^3^ ≥2 chronic conditions

^4^ ≥1 on activities of daily living scale

^5^ ≥4 on EURO-Depression scale

^6^ As used in Cheval et al., 2018, Med Sci Sports Exerc [34], 2 variables were used to assess the level of daily life physical activity. The first item assessed vigorous physical activity (“How often do you engage in vigorous physical activity, such as sports, heavy housework, or a job that involves physical labour?”). The second item assessed moderate physical activity (“How often do you engage in activities that require a low or moderate level of energy such as gardening, cleaning the car, or doing a walk?”). Participants answered by using a 4-point scale (1, >1 a week; 2, once a week; 3, 1–3 times a month; 4, hardly ever, or never). Participants who did not answer “1” to either question were classified as “physically inactive”.

### Multimorbidity

#### Childhood SECs (CSCs)

The odds of multimorbidity increased with age (OR = 3·47 for each 10 years, 95%CI 3·15–3·82) (Table 2). All CSC categories were associated with odds of multimorbidity [Model 1, reference: most disadvantaged; disadvantaged: OR = 0.79 (95%CI 0.70–0.90), middle: OR = 0.60 (95%CI 0.53–0.68), advantaged: OR = 0.52 (95%CI 0.45–0.60), most advantaged: OR = 0.40 (95%CI 0.34–0.48)]. The association between CSCs and multimorbidity remained statistically significant after adjusting for ACSs (Model 2) and risk factors (Model 3), except for disadvantaged. In all models, we observed a gradient across the ORs of CSC categories, which shows that older adults with more advantaged CSCs had lower odds of multimorbidity than older adults with the most disadvantaged CSCs.

**Table 2:** Association of childhood socioeconomic conditions (CSCs) with odds of multimorbidity at age 73 years. (N = 31,432).

	Model 1		Model 2		Model 3	
*Variables*	*OR (95% CI)*	*P-value*	*OR (95% CI)*	*P-value*	*OR (95% CI)*	*P-value*
Linear age (10-year follow-up)	3·47 (3·15–3·82)	<0·001	3·53 (3·21–3·89)	<0·001	3·36 (3·06–3·69)	<0·001
Squared age (10-year follow-up)	0·89 (0·85–0·93)	<0·001	0·88 (0·84–0·92)	<0·001	0·89 (0·85–0·93)	<0·001
*Sex (ref*. *women)*	0·83 (0·78–0·90)	<0·001	0·88 (0·82–0·95)	0·001	0·87 (0·81–0·93)	<0·001
*CSCs (ref*. *most disadvantaged)*						
Disadvantaged	0·79 (0·70–0·90)	<0·001	0·89 (0·78–1·01)	0·061	0·89 (0·79–1·01)	0·066
Middle	0·60 (0·53–0·68)	<0·001	0·77 (0·68–0·87)	<0·001	0·79 (0·70–0·90)	<0·001
Advantaged	0·52 (0·45–0·60)	<0·001	0·74 (0·64–0·85)	<0·001	0·77 (0·68–0·89)	<0·001
Most advantaged	0·40 (0·34–0·48)	<0·001	0·66 (0·55–0·80)	<0·001	0·70 (0·58–0·83)	<0·001
*Education (ref*. *primary)*						
Secondary	-		0·67 (0·60–0·74)	<0·001	0·72 (0·65–0·80)	<0·001
Tertiary	-		0·47 (0·41–0·54)	<0·001	0·54 (0·47–0·62)	<0·001
*Main occupation (ref*. *high skill)*						
Low skill	-		0·86 (0·78–0·94)	0·001	0·89 (0·82–0·97)	0·006
Never worked	-		0·98 (0·81–1·18)	0·809	0·98 (0·81–1·18)	0·814
*Financial strain (able to make ends meet) (ref*. *easily)*						
Fairly easily	-		1·13 (1·06–1·20)	<0·001	1·10 (1·04–1·17)	0·002
Some difficulty	-		1·48 (1·37–1·60)	<0·001	1·42 (1·31–1·53)	<0·001
Great difficulty	-		2·05 (1·83–2·29)	<0·001	1·95 (1·74–2·18)	<0·001

*Notes*: OR = odds ratios; CI = confidence interval. All models were adjusted for birth cohorts, attrition, and countries. Model 3 additionally adjusted for obesity, alcohol consumption (at baseline), smoking (at baseline) and physical activity.

The analyses stratified by sex (S7 Table in S1 File) showed an association between CSC categories and odds of multimorbidity for both sexes (except for the disadvantaged CSC category for men, Model 1a). However, after adjusting for ACSs, CSCs did not remain associated with multimorbidity for men (Model 2a). However, for women, CSCs remained associated with multimorbidity (Models 1a, 2a and 3a).

#### Evolution of odds of multimorbidity during ageing depending on CSCs

We did not find evidence of an interaction between age (linear and quadratic) and CSCs on multimorbidity (Models 1 to 3; S3 Table in S1 File). In the analyses stratified by sex (S7 Table in S1 File), results were consistent with those of the main analysis, with no evidence of an interaction between age (linear and quadratic) and CSCs on multimorbidity in both sexes. However, for women, the most advantaged CSCs the rate of increase in odds was reduced in the model adjusted for ACSs (age*most advantaged, OR = 0·66, 95%CI 0·49–0·90, reference category: most disadvantaged women, Model 2b), which remained significant after adjusting for risk factors (Model 3b).

#### Life-course score

We found a significant association between the life-course score and odds of multimorbidity (Table 3, Model 1, Table 3: OR = 0·74 per unit-increase in the life-course score, 95%CI 0·73–0·76), which remained significant after adjusting for risk factors (Model 2: OR = 0·78, 95%CI 0·76–0·80). A higher score, representing more advantaged life-course SECs, was associated with reduced odds of multimorbidity. In the analyses stratified by sex, we found a significant association between the life-course score and odds of multimorbidity for women and men (S8 Table in S1 File, Model 1a; women: OR = 0·71, 95%CI 0·68–0·73; men: OR = 0·78, 95%CI 0·76–0·81). These associations remained significant after adjusting for risk factors (Model 2a, women: OR = 0·75, 95%CI 0·72–0·77; men: OR = 0·81, 95%CI 0·79–0·84).

**Table 3:** Association of life-course socioeconomic circumstances (SECs) score with odds of multimorbidity at age 73 years. (N = 31,432).

	Model 1		Model 2	
*Variables*	*OR (95% CI)*	*P-value*	*OR (95% CI)*	*P-value*
Linear age (10-year follow-up)	3·44 (3·12–3·78)	<0·001	3·28 (2·99–3·61)	<0·001
Squared age (10-year follow-up)	0·88 (0·84–0·92)	<0·001	0·89 (0·85–0·93)	<0·001
Sex (ref. women)	0·91 (0·85–0·98)	0·01	0·89 (0·83–0·96)	0·001
Life-course score (per-unit increase)^a^	0·74 (0·73–0·76)	<0·001	0·78 (0·76–0·80)	<0·001

*Notes*: OR = odds ratios; CI = confidence interval. All models were adjusted for birth cohorts, attrition and countries. Model 2 additionally adjusted for obesity, alcohol consumption (at baseline), smoking (at baseline) and physical activity.

^a^ Range of the life-course score: 0 to 8. A higher life-course score means a longer life-time exposure to advantaged SECs during the entire life course.

#### Evolution of the odds of multimorbidity during ageing depending on life-course SECs

We found a borderline significant association between linear age and life-course score (Model 1: OR = 1·03, 95%CI 1·01–1·05), which became not significant after adjusting for risk factors (Model 2: OR = 1·02, 95%CI 0·99–1·04) (S4 Table in S1 File). In the analyses stratified by sex (S8 Table in S1 File), we found non-significant interactions between the life-course score and age, so life-course SECs did not modify the increased odds of multimorbidity during ageing (Models 1b and 2b).

### Polypharmacy

The odds of polypharmacy increased linearly with age (Table 4, Model 3: OR = 2·14, 95%CI 1·78–2·57). The odds of polypharmacy with disadvantaged CSCs did not significantly differ from the odds with advantaged CSCs (Model 1, reference category: most disadvantaged; disadvantaged: OR = 0.97, 95% 0.70–1.34; middle: OR = 0.81, 95%CI 0.58–1.12; advantaged: OR = 0.73, 95% 0.51–1.04, most advantaged: OR = 0.68, 95%CI 0.42–1.09), regardless of adjustment for ACSs (Model 2) or polypharmacy risk factors (Model 3). The analyses stratified by sex gave similar results (S9 Table in S1 File). For men, the odds of polypharmacy were not significantly associated with advantaged CSCs (Model 1a), regardless of the adjustment for ACSs (Model 2a) and risk factors (Model 3a). For women, CSCs were associated with the odds of polypharmacy but did not remain after adjusting for ACSs (Model 2a).

**Table 4:** Association of childhood socioeconomic conditions (CSCs) with odds of polypharmacy at age 73 years. (N = 21,794).

	Model 1		Model 2		Model 3	
**Variables**	*OR (95% CI)*	*P-value*	*OR (95% CI)*	*P-value*	*OR (95% CI)*	*P-value*
Linear age (10-year follow-up)	4·09 (3·03–5·51)	<0·001	4·15 (3·08–5·60)	<0·001	2·14 (1·78–2·57)	<0·001
Squared age (10-year follow-up)	1·29 (1·11–1·50)	0·001	1·28 (1·10–1·49)	0·001	1·00 (0·91–1·09)	0·946
*Sex (ref*. *women)*	1·12 (0·92–1·36)	0·259	1·18 (0·96–1·43)	0·112	1·40 (1·25–1·58)	<0·001
*CSCs (ref*. *most disadvantaged)*						
Disadvantaged	0·97 (0·70–1·34)	0·835	0·99 (0·71–1·38)	0·961	1·01 (0·84–1·22)	0·900
Middle	0·81 (0·58–1·12)	0·205	0·91 (0·65–1·28)	0·592	0·98 (0·81–1·18)	0·799
Advantaged	0·73 (0·51–1·04)	0·082	0·86 (0·59–1·25)	0·429	0·97 (0·79–1·20)	0·793
Most advantaged	0·68 (0·42–1·09)	0·111	0·85 (0·51–1·40)	0·512	0·91 (0·69–1·21)	0·529
*Education (ref*. *primary)*						
Secondary	-	-	0·75 (0·56–1·00)	0·047	0·80 (0·68–0·94)	0·006
Tertiary	-	-	0·66 (0·46–0·96)	0·03	0·76 (0·62–0·93)	0·009
*Main occupation (ref*. *high skill)*						
Low skill	-	-	1·08 (0·85–1·38)	0·506	1·03 (0·90–1·18)	0·673
Never worked	-	-	1·22 (0·70–2·13)	0·477	1·10 (0·81–1·50)	0·548
*Financial strain (able to make ends meet) (ref*. *easily)*						
Fairly easily	-	-	1·19 (1·00–1·42)	0·054	1·17 (1·04–1·31)	0·008
Some difficulty	-	-	1·37 (1·11-·1·69)	0·003	1·34 (1·17–1·53)	<0·001
Great difficulty	-	-	1·93 (1·42–2·63)	<0·001	1·74 (1·43–2·11)	<0·001

*Notes*: OR = odds ratios; CI = confidence interval; All models were adjusted for birth cohorts, squared age, and countries. Model 3 additionally adjusted for obesity, alcohol consumption (at baseline), smoking (at baseline), physical activity, multimorbidity, depression, limitation with activities of daily activity and living in a nursing home.

#### Evolution of the odds of polypharmacy during ageing depending on CSCs

For both men and women, the different CSC categories did not modify the increased odds of polypharmacy during ageing (S5 and S9 Tables in S1 File).

#### Life-course score

We found a significant association between the life-course score and odds of polypharmacy (Table 5), before adjusting for risk factors (OR = 0·81 per-unit increase in life-course score, 95%CI 0·69–0·96) and after (OR = 0·86, 95%CI 0·83–0·90). We found a significant association between the life-course score and odds of polypharmacy for women (S10 Table in S1 File, Model 1a, women: OR = 0·75, 95%CI 0·69–0·83) but not men (OR = 0·86, 95%CI 0·68–1·09). For women, this association remained significant after adjusting for risk factors (Model 2a, OR = 0·81, 95%CI 0·77–0·86). For men, the fully adjusted model showed decrease odds of polypharmacy with increasing life-course score, but the lower odds were less pronounced than for women (OR = 0·92, 95%CI 0·87–0·96).

**Table 5:** Association of life-course SECs score with odds of polypharmacy at age 73 years. (N = 21,794).

	Model 1		Model 2	
*Variables*	*OR (95% CI)*	*P-value*	*OR (95% CI)*	*P-value*
Linear age (10-year follow-up)	2·84 (1·85–4·35)	<0·001	2·10 (1·75–2·52)	<0·001
Squared age (10-year follow-up)	1·00 (0·79–1·26)	0·997	1·00 (0·92–1·09)	0·982
Sex (ref. women)	1·16 (0·69–1·96)	0·578	1·41 (1·26–1·58)	<0·001
Life-course score (per-unit increase) ^a^	0·81 (0·69–0·96)	0·012	0·86 (0·83–0·90)	<0·001

*Notes*: OR = odds ratios; CI = confidence interval; SEC = socioeconomic conditions. All models were adjusted for birth cohorts, squared age, and countries. Model 2 additionally adjusted for obesity, alcohol consumption (at baseline), smoking (at baseline), physical activity, multimorbidity, depression, limitation with activities of daily activity and living in a nursing home.

^a^ Range of the life-course score: 0–8, with 0 indicating a disadvantaged life course and 8 an advantaged life course. A higher score means longer life-time exposure to advantaged SECs during the entire life course

#### Evolution of the odds of polypharmacy during ageing depending on life-course SECs

Life-course SECs did not modify the odds of polypharmacy during ageing (S10 Table in S1 File, Models 1b and 2b).

#### Robustness and sensitivity analyses

Results from sensitivity and robustness analyses are reported in S11 Table in S1 File. Overall, results from sensitivity analyses and robustness analyses (applied to multimorbidity only) were similar to those of the main analysis. However, one exception was when including the category “other chronic conditions” in the score of multimorbidity. When considering the association with the life-course score, we observed a significant interaction between linear age and life-course score, in both the minimally adjusted (Model 1) and fully adjusted (Model 2) models. Interactions with age square were not significant. This result suggested that the (intra-individual) change during ageing in the risk of multimorbidity was quicker (linear increase, not accelerated) for participants with longer life-time exposure to advantaged SECs. Because participants with life-course socioeconomic advantages had lower odds of multimorbidity, they may have a higher rate of increase in odds of multimorbidity following a phenomenon of adjustment during ageing (reduction of differences by survivor selection). Also, the effect of longer life-time exposure to socioeconomic advantages may have a protective effect similar to the process of cognitive reserve [39]: life-time socioeconomic advantages may protect against the onset of multimorbidity symptomatology, but such protection may be reduced in old age.

## Discussion

We investigated whether disadvantaged CSCs predict increased odds of multimorbidity and polypharmacy in older adults, whether CSCs remain associated when adjusting for ACSs, and whether CSCs and ACSs are associated cumulatively over the life course. We found CSCs associated with odds of multimorbidity in older adults. All categories of the CSCs were associated with odds of multimorbidity, with higher advantage leading to lower odds of multimorbidity. We also observed a gradient across CSC category estimates. When adjusting for ACSs, the association with CSCs was attenuated but remained significant. Notably, this result was robust across various sensitivity and robustness analyses. We found no variation in the evolution of odds during individual ageing. However, women with more advantaged CSCs showed less increase in odds of multimorbidity as they aged as compared with women with disadvantaged CSCs. We also found an association between the life-course score and multimorbidity: longer experience with advantaged SECs during the lifetime was associated with less odds of multimorbidity in older age, a result that was robust to adjustment for risk factors.

We did not find an association between CSCs and polypharmacy after adjusting for ACSs, and CSCs did not modify the rate of increased odds of polypharmacy during ageing. For women but not men, the odds of polypharmacy decreased with a more advantaged life-course score. This is an important finding because to the best of our knowledge, this is the first study to examine the association between polypharmacy and life-course factors.

Previous research also found an association between factors such as financial hardship during childhood, education, or childhood health status and multimorbidity in old age [10, 40, 41]. One study found that the effect of childhood socioeconomic status disappeared after adjusting for adulthood health status and SECs [10]. Conversely, in our analyses stratified by sex, the association between CSCs and multimorbidity in older age seemed to disappear in men after adjusting for ACSs but persisted in women. Even after adjusting for ACSs, a gradient was observed: women with less advantaged CSCs remained with increased odds of multimorbidity than women with more advantaged CSCs. For men, more advantaged SECs during adulthood may have compensated a disadvantaged start in life, a result previously observed in a study of disability [42]. However, this does not seem to hold true for women and must be interpreted with caution. This finding could simply be an artefact of survivorship bias, since men are more likely to die at a younger age than women. Hence, those who survive into later life are more likely to be healthier individuals. These findings might also be generational and reflect gender inequalities in access to education and employment between men and women, since most of our study participants were born in the first half of the 20th century. During this period, men had more opportunities than women to improve their socio-economic living conditions in the society of that time.

Our finding of an association between the life-course score and multimorbidity agrees with previous research, which also found that the longer the experience with advantaged SECs during the lifetime, the less likely is multimorbidity. For instance, one study looked at lifetime savings and found higher lifetime earnings associated with lower number of chronic conditions [40]. Another study using a score of paternal occupation in childhood and own adult education found an association between life-course social position and risk of type 2 diabetes; with lower social positions associated with increased odds of the disease [42]. Our approach adds that the association is multidimensional and incorporates several variables that capture different dimensions of CSCs and ACSs, which together depict a clearer picture of the life-course.

For women, CSCs affected the rate of increase in odds of multimorbidity for those born in the most advantaged households. The more advantaged the CSCs, the slower the increase in odds of multimorbidity during ageing. This effect was not affected by adjustment for multimorbidity risk factors. Other studies have found similar results, for instance, that secondary and tertiary education and life-long occupations reduced the speed of multimorbidity development over time [43]. These associations suggest that efforts to prevent multimorbidity and its development during ageing should consider CSCs and ASCs of patients, especially in women.

We did not find an association of CSCs and polypharmacy. However, we found an association between the life-course score and polypharmacy, which was not confounded by polypharmacy risk factors. In other words, the longer the exposure to disadvantaged SECs over the life course, the greater the odds of polypharmacy in old age. These results do not support the critical-period hypothesis; however, they support the accumulation-of-exposures hypothesis. Because of the lack of previous studies on this topic, we cannot compare our findings to previous study results. Nevertheless, our findings are important in the context of the potentially inappropriate medication use that often accompanies polypharmacy [44].

We see different reasons why the two cohorts have different sample sizes and why the results for multimorbidity and polypharmacy were different. First, while multimorbidity is often accompanied by polypharmacy, it not always does since not every patient with ≥2 chronic conditions regularly uses ≥5 regular medications [45, 46]. This is due to the fact that there are also non-pharmacological treatment options for some chronic conditions (e.g., low calorie diet, physical activity, and weight loss for diabetes). Second, participants may have stated to have polypharmacy, but not have stated to have ≥2 chronic conditions from the list of chronic conditions in the SHARE questionnaires. Third, the way in which polypharmacy was collected in the SHARE study is broad, as it includes prescription drugs, over-the-counter medications, and dietary supplements. This means that participants using ≥5 supplements and/or over-the-counter medications, but not having any prescription medications to treat diagnosed chronic conditions, were labelled as having polypharmacy. They thus may have been selected into the polypharmacy cohort, while at the same time not having been selected into the multimorbidity cohort. Fourth, the two cohorts were defined based on the available data on multimorbidity and polypharmacy in the SHARE database. In SHARE, data on multimorbidity is collected at each wave while data on polypharmacy was only collected in the last two waves. This short duration of follow-up (2 waves, i.e. 2 years) can account for the null results observed for the evolution of odds of polypharmacy during ageing.

The strengths of our study include the large sample size, the use of multidimensional measures of childhood and ACSs, the capacity to comprehensively adjust our models for risk factors associated with the outcome, and the creation of a life-course score. Our study has several limitations. First, all the information used in our analyses was self-reported (except for the cognitive performance tests) and information on childhood and ACSs was collected retrospectively, which might have implied recall bias. However, extensive literature has shown that this bias was minor [47]. Second, the assessment of chronic conditions in the SHARE study was based on a list of 20 conditions, which might have led to the misclassification of patients with chronic conditions not listed in the data collection tool. Self-reported information on chronic conditions is often lower than the information found in administrative data [48], which may have led to an underestimation of participants with multimorbidity. Third, potential selection bias cannot be excluded because participants died or dropped out during the follow-up period, and we excluded participants who did not participate in the SHARELIFE module (wave 3 or 7). However, we reduced the selection bias by including participants with only one assessment during follow-up and by adjusting all models with participant attrition. Furthermore, because polypharmacy was assessed only in the last 2 waves of SHARE, this bias was less relevant for polypharmacy than multimorbidity analyses. Fourth, we built an unvalidated score of life-course SECs, which gave the same weight to each of the life-course periods. Fifth, we performed an associations study and did not frame our analyses within contemporary causal inference methods. We discuss causality implications in S18 Appendix in S1 File. Finally, the SHARE questionnaire did not assess whether the polypharmacy was long-term.

In summary, childhood socioeconomic factors were associated with odds of multimorbidity in older age in participants across 17 countries. Despite some differences between men and women, in general, more advantaged SECs during the entire life course led to reduced odds of multimorbidity and polypharmacy. Men but not women seem to be able to compensate for a disadvantaged start in life with more advantaged SECs during adulthood. This information should be used in efforts to prevent multimorbidity and optimize polypharmacy and care in older adults (e.g., screen for adults at higher risk of multimorbidity and polypharmacy). This supports a shift from the management of multimorbidity in old age towards multimorbidity prevention throughout the life course. Public health would benefit from adopting a life-course perspective in the training of healthcare professionals [49]. Same as for dementia [50], health promotion should start early in life to prevent multimorbidity in old age [51].

## Supporting information

S1 Checklist STROBE checklist. (DOCX)

S1 File (DOCX)
