# Correction: Life-course socioeconomic conditions, multimorbidity and polypharmacy in older adults: A retrospective cohort study

**DOI:** 10.1371/journal.pone.0305198

**Published:** 2024-06-04

**Authors:** The PLOS One Staff

The fourth author’s initials appear incorrectly in the citation. The correct citation is: Jungo KT, Cheval B, Sieber S, van der Linden BWA, Ihle A, Carmeli C, et al. (2022) Life-course socioeconomic conditions, multimorbidity and polypharmacy in older adults: A retrospective cohort study. PLoS ONE 17(8): e0271298. https://doi.org/10.1371/journal.pone.0271298.

The publisher apologizes for the error.
