# Supplementary material for: Life-course socioeconomic conditions, multimorbidity and polypharmacy in older adults: A retrospective cohort study
Source: PLoS One. 2022 Aug 2;17(8):e0271298. doi: 10.1371/journal.pone.0271298 (PMC9345356; doi:10.1371/journal.pone.0271298)
Supplement: S1 File — (DOCX) [file pone.0271298.s002.docx]

**S1 Figure.** Flow chart of participant inclusion: Cohort for multimorbidity analyses.

Excluded due to not having information on all covariates
(n = 21,044)

Excluded due to not having information adulthood socioeconomic conditions
(n = 19,399)

Excluded due to not having information childhood socioeconomic conditions
(n = 62,811)

SHARE participants with information on chronic conditions

(N = 134,686)

Criterion 2: Participants with information on adulthood socioeconomic conditions
(n = 52,476)

Criterion 1: Participants with information on childhood socioeconomic conditions
(n = 71,875)

Participants with information on all covariates

(N = 31,432)

**S2 Figure.** Flow chart of participant inclusion: Cohort for polypharmacy analyses.

Participants having information on all covariates

(n = 21,794)

Criterion 1: Participants with information on childhood socioeconomic conditions
(n = 50,751)

Criterion 2: Participants with information on adulthood socioeconomic conditions
(n = 36,181)

SHARE participants with information on polypharmacy

(N = 74,017)

Excluded due to not having information adulthood socioeconomic conditions
(n = 14,570)

Excluded due to not having information on all covariates
(n = 14,387)

Excluded due to not having information childhood socioeconomic conditions
(n = 23,266)

| **S3 Table.** Association of childhood socioeconomic conditions (CSCs) with odds of multimorbidity at age 73 years and during ageing. (N = 31,432) | | | | | | | |
| --- | --- | --- | --- | --- | --- | --- | --- |
|  | **Model 1** |  | **Model 2** |  | **Model 3** |  |  |
| **Variables** | OR (95% CI) | P-value | OR (95% CI) | P-value | OR (95% CI) | P-value |  |
| *Linear age (10-year follow-up)* | 3·25 (2·83-3·73) | <0·001 | 3·07 (2·61-3·60) | <0·001 | 3·20 (2·66-3·86) | <0·001 |  |
| *Squared age (10-year follow-up)* | 0·85 (0·77-0·95) | 0·003 | 0·89 (0·79-1·01) | 0·075 | 0·89 (0·77-1·04) | 0·143 |  |
| *Sex (ref. women)* | 0·83 (0·78-0·90) | <0·001 | 0·88 (0·82-0·94) | <0·001 | 0·86 (0·80-0·93) | <0·001 |  |
| *CSCs (ref. most disadvantaged)* | | | | | | | |
| Disadvantaged | 0·77 (0·66-0·90) | 0·001 | 0·84 (0·72-0·99) | 0·034 | 0·86 (0·74-1·01) | 0·061 |  |
| Middle | 0·57 (0·49-0·67) | <0·001 | 0·71 (0·60-0·83) | <0·001 | 0·75 (0·64-0·87) | <0·001 |  |
| Advantaged | 0·52 (0·44-0·61) | <0·001 | 0·70 (0·59-0·84) | <0·001 | 0·74 (0·63-0·88) | 0·001 |  |
| Most advantaged | 0·41 (0·32-0·51) | <0·001 | 0·62 (0·49-0·79) | <0·001 | 0·66 (0·52-0·83) | <0·001 |  |
| *Education (ref. primary)* | | | | | | | |
| Secondary | - | - | 0·68 (0·60-0·78) | <0·001 | 0·73 (0·65-0·84) | <0·001 |  |
| Tertiary | - | - | 0·50 (0·42-0·59) | <0·001 | 0·56 (0·47-0·66) | <0·001 |  |
| *Main occupation (ref. high skill)* |  | | | | | | |
| Low skill | - | - | 0·95 (0·84-1·07) | 0·37 | 0·98 (0·88-1·10) | 0·75 |  |
| Never worked | - | - | 1·00 (0·79-1·28) | 0·97 | 1·00 (0·79-1·27) | 0·982 |  |
| *Financial strain (able to make ends meet)* | | | | | | | |
| Fairly easily | - | - | 1·18 (1·09-1·28) | <0·001 | 1·17 (1·08-1·27) | <0·001 |  |
| Some difficulty | - | - | 1·46 (1·32-1·61) | <0·001 | 1·40 (1·27-1·55) | <0·001 |  |
| Great difficulty | - | - | 1·94 (1·66-2·26) | <0·001 | 1·87 (1·61-2·18) | <0·001 |  |
| Age*CSC |  | | | | | |  |
| Age*Disadvantaged | 1·04 (0·90-1·20) | 0·589 | 1·01 (0·88-1·16) | 0·873 | 1·02 (0·89-1·17) | 0·787 |  |
| Age*Middle | 1·14 (0·99-1·30) | 0·069 | 1·05 (0·91-1·21) | 0·494 | 1·05 (0·91-1·20) | 0·5 |  |
| Age*Advantaged | 1·11 (0·95-1·29) | 0·189 | 1·00 (0·85-1·18) | 0·981 | 1·00 (0·86-1·16) | 0·979 |  |
| Age*Most advantaged | 1·03 (0·85-1·25) | 0·738 | 0·89 (0·72-1·09) | 0·269 | 0·91 (0·75-1·11) | 0·366 |  |
| Age2*CSC |  | | | | | |  |
| Age2*Disadvantaged | 1·03 (0·91-1·16) | 0·655 | 1·03 (0·92-1·17) | 0·587 | 1·02 (0·91-1·15) | 0·728 |  |
| Age2*Middle | 1·09 (0·97-1·22) | 0·15 | 1·10 (0·97-1·24) | 0·137 | 1·08 (0·96-1·21) | 0·226 |  |
| Age2*Advantaged | 1·03 (0·91-1·17) | 0·596 | 1·05 (0·92-1·19) | 0·498 | 1·03 (0·91-1·17) | 0·658 |  |
| Age2*Most advantaged | 0·99 (0·85-1·16) | 0·936 | 1·01 (0·86-1·19) | 0·927 | 1·00 (0·86-1·18) | 0·953 |  |
| Age*Education |  | | | | | |  |
| Age*Secondary | - | - | 1·19 (1·06-1·33) | 0·003 | 1·15 (1·03-1·28) | 0·015 |  |
| Age*Tertiary | - | - | 1·27 (1·08-1·49) | 0·003 | 1·21 (1·04-1·41) | 0·014 |  |
| Age2*Education |  | | | | | |  |
| Age2*Secondary | - | - | 0·99 (0·90-1·09) | 0·807 | 0·99 (0·90-1·09) | 0·834 |  |
| Age2*Tertiary | - | - | 0·99 (0·90-1·09) | 0·807 | 0·99 (0·90-1·09) | 0·834 |  |
| Age*Main occupation |  | | | | | |  |
| Age*Low skill | - | - | 1·06 (0·95-1·19) | 0·292 | 1·05 (0·94-1·17) | 0·389 |  |
| Age*Never worked | - | - | 1·14 (0·94-1·38) | 0·175 | 1·11 (0·92-1·34) | 0·268 |  |
| Age2*Main occupation |  | | | | | |  |
| Age2*Low skill | - | - | 0·93 (0·86-1·02) | 0·112 | 0·93 (0·86-1·02) | 0·109 |  |
| Age2*Never worked | - | - | 0·98 (0·83-1·15) | 0·801 | 0·98 (0·84-1·16) | 0·851 |  |
| Age*Satisfaction with income |  | | | | | |  |
| Age*Fairly easily | - | - | 0·93 (0·86-1·01) | 0·077 | 0·94 (0·87-1·01) | 0·084 |  |
| Age*Some difficulty | - | - | 0·94 (0·82-1·09) | 0·42 | 0·96 (0·84-1·11) | 0·595 |  |
| Age*Great difficulty | - | - | 0·99 (0·90-1·09) | 0·854 | 1·00 (0·91-1·09) | 0·955 |  |
| Age2*Satisfaction with income |  | | | | | |  |
| Age2*Fairly easily | - | - | 0·92 (0·86-0·98) | 0·015 | 0·92 (0·86-0·98) | 0·01 |  |
| Age2*Some difficulty | - | - | 1·01 (0·90-1·13) | 0·902 | 1·00 (0·89-1·12) | 0·977 |  |
| Age2*Great difficulty | - | - | 1·00 (0·93-1·09) | 0·901 | 1·01 (0·93-1·09) | 0·824 |  |
| *Legend*: OR=odds ratios; CI=confidence interval; All models were adjusted for birth cohorts, attrition and countries. Model 2 additionally adjusted for obesity, alcohol consumption (at baseline), smoking (at baseline) and physical activity. | | | | | | | |

| **S4 Table.** Association of life-course socioeconomic conditions (SECs) score with odds of multimorbidity at age 73 years and during ageing. (N = 31,432) | | | | |
| --- | --- | --- | --- | --- |
|  | **Model 1** |  | **Model 2** |  |
| Variables | OR (95% CI) | P-value | OR (95% CI) | P-value |
| Linear age (10-year follow-up) | 3·02 (2·63-3·47) | <0·001 | 3·29 (2·77-3·92) | <0·001 |
| Squared age (10-year follow-up) | 0·93 (0·84-1·02) | 0·119 | 0·93 (0·82-1·06) | 0·307 |
| Sex (ref. women) | 0·91 (0·84-0·97) | 0·008 | 0·89 (0·83-0·95) | 0·001 |
| Obesity | - | - | 2·70 (2·45-2·98) | <0·001 |
| High alcohol consumption | - | - | 1·02 (0·91-1·14) | 0·745 |
| Low physicial activity | - | - | 1·32 (1·22-1·43) | <0·001 |
| Smoking | - | - | 0·93 (0·83-1·05) | 0·235 |
| Life-course score | 0·76 (0·74-0·78) | <0·001 | 0·80 (0·78-0·82) | <0·001 |
| Age*Life-course score | 1·03 (1·01-1·05) | 0·017 | 1·02 (0·99-1·04) | 0·154 |
| Age2*Life-course score | 0·99 (0·97-1·01) | 0·278 | 0·99 (0·97-1·01) | 0·214 |
| Age*Obesity | - | - | 0·74 (0·66-0·82) | <0·001 |
| Age2*Obesity | - | - | 0·94 (0·87-1·02) | 0·135 |
| Age*High alcohol use | - | - | 0·94 (0·84-1·06) | 0·331 |
| Age2*High alcohol use | - | - | 1·00 (0·92-1·09) | 0·978 |
| Age*Low physical activity | - | - | 1·01 (0·94-1·08) | 0·826 |
| Age2*Low physical activity | - | - | 0·99 (0·93-1·05) | 0·743 |
| Age*Smoking | - | - | 0·86 (0·75-0·99) | 0·039 |
| Age2*Smoking | - | - | 1·04 (0·94-1·14) | 0·453 |
|  |  |  |  |  |
| *Legend*: OR=odds ratios; CI=confidence interval. All models were adjusted for birth cohorts, attrition and countries. Model 2 additionally adjusted for obesity, alcohol consumption (at baseline), smoking (at baseline) and physical activity. | | | | |

| **S5 Table.** Association of CSCs with odds of polypharmacy at age 73 years and during ageing. (N = 21,794) | | | | | | |
| --- | --- | --- | --- | --- | --- | --- |
|  | **Model 1** |  | **Model 2** |  | **Model 3** |  |
| **Variables** | OR (95% CI) | P-value | OR (95% CI) | P-value | OR (95% CI) | P-value |
| *Linear age (10-year follow-up)* | 2·78 (1·84-4·20) | <0·001 | 2·64 (1·51-4·61) | 0.001 | 2·65 (1·88-3·74) | <0·001 |
| *Squared age (10-year follow-up)* | 1·19 (0·87-1·63) | 0·275 | 1·13 (0·71-1·80) | 0.595 | 1·01 (0·76-1·33) | 0·966 |
| *Sex (ref. women)* | 1·11 (0·92-1·35) | 0·278 | 1·17 (0·96-1·43) | 0.127 | 1·24 (1·10-1·39) | <0·001 |
| *CSCs (ref. most disadvantaged)* | | | | | | |
| Disadvantaged | 0·93 (0·62-1·40) | 0·74 | 1·00 (0·66-1·51) | 0.999 | 1·08 (0·85-1·38) | 0·537 |
| Middle | 0·78 (0·53-1·17) | 0·233 | 0·88 (0·58-1·33) | 0.531 | 1·17 (0·92-1·49) | 0·193 |
| Advantaged | 0·74 (0·48-1·15) | 0·184 | 0·87 (0·55-1·38) | 0.551 | 1·27 (0·97-1·65) | 0·082 |
| Most advantaged | 0·62 (0·34-1·11) | 0·11 | 0·78 (0·42-1·45) | 0.428 | 1·13 (0·78-1·62) | 0v523 |
| *Education (ref. primary)* | | | | | | |
| Secondary | - | - | 0·76 (0·53-1·07) | 0.119 | 0·81 (0·67-0·99) | 0·038 |
| Tertiary | - | - | 0·75 (0·47-1·18) | 0.208 | 0·84 (0·65-1·09) | 0·195 |
| *Main occupation (ref. high skill)* | | | | | | |
| Low skill | - | - | 1·13 (0·83-1·53) | 0.439 | 1·03 (0·86-1·23) | 0·745 |
| Never worked | - | - | 1·19 (0·60-2·36) | 0.616 | 0·68 (0·45-1·01) | 0·056 |
| *Financial strain (able to make ends meet)* | | | | | | |
| Fairly easily | - | - | 1·16 (0·92-1·45) | 0.211 | 1·04 (0·90-1·21) | 0·603 |
| Some difficulty | - | - | 1·37 (1·05-1·79) | 0.021 | 1·02 (0·86-1·21) | 0·832 |
| Great difficulty | - | - | 1·74 (1·17-2·59) | 0.007 | 1·02 (0·79-1·32) | 0·88 |
| *Obesity* | - | - | - | - | 2·89 (2·45-3·42) | <0·001 |
| *High alcohol consumption* | - | - | - | - | 1·09 (0·92-1·30) | 0·334 |
| *Low physicial activity* | - | - | - | - | 1·63 (1·42-1·88) | <0·001 |
| *Smoking* | - | - | - | - | 0·99 (0·83-1·19) | 0·953 |
| *Multimorbidity* | - | - | - | - | 5·99 (5·25-6·85) | <0·001 |
| *Depression* | - | - | - | - | 1·95 (1·68-2·26) | <0·001 |
| *ADL* | - | - | - | - | 3·17 (2·55-3·94) | <0·001 |
| *Living situation (nursing home)* | - | - | - | - | 4·59 (0·79-26·76) | 0·09 |
| Age*CSCs |  | | | | | |
| Age*Disadvantaged | 1·22 (0·82-1·80) | 0·328 | 1·04 (0·70-1·56) | 0·847 | 1·03 (0·82-1·30) | 0·769 |
| Age*Middle | 1·17 (0·80-1·71) | 0·42 | 1·00 (0·66-1·49) | 0·981 | 0·97 (0·77-1·22) | 0.785 |
| Age*Advantaged | 1·20 (0·79-1·81) | 0·395 | 1·00 (0·64-1·56) | 0·993 | 0·97 (0·75-1·25) | 0·804 |
| Age*Most advantaged | 1·05 (0·62-1·77) | 0·851 | 0·74 (0·42-1·31) | 0·302 | 0·71 (0·51-0·99) | 0·041 |
| Age2*CSCs | | | | | | |
| Age2*Disadvantaged | 0·98 (0·68-1·42) | 0·912 | 0·94 (0·64-1·37) | 0·755 | 1·00 (0·80-1·24) | 0·981 |
| Age2*Middle | 1·00 (0·70-1·41) | 0·982 | 1·03 (0·71-1·49) | 0·893 | 1·03 (0·83-1·28) | 0·788 |
| Age2*Advantaged | 0·95 (0·65-1·38) | 0·793 | 0·99 (0·66-1·48) | 0·956 | 0·99 (0·79-1·25) | 0·925 |
| Age2*Most advantaged | 0·99 (0·63-1·56) | 0·97 | 1·00 (0·62-1·64) | 0·986 | 1·02 (0·77-1·35) | 0·913 |
| Age*Education | | | | | | |
| Age*Secondary | - | - | 1·10 (0·81-1·51) | 0·538 | 1·10 (0·92-1·32) | 0·276 |
| Age*Tertiary | - | - | 1·24 (0·80-1·92) | 0·334 | 1·21 (0·94-1·54) | 0·133 |
| Age2*Education | | | | | | |
| Age2*Secondary | - | - | 1·00 (0·75-1·34) | 0·983 | 1·02 (0·86-1·20) | 0·849 |
| Age2*Tertiary | - | - | 0·94 (0·63-1·39) | 0·748 | 0·98 (0·79-1·22) | 0·85 |
| Age*Main occupation | | | | | | |
| Age*Low skill | - | - | 0·93 (0·69-1·25) | 0·621 | 0·80 (0·68-0·95) | 0·01 |
| Age*Never worked | - | - | 1·07 (0·57-1·99) | 0·836 | 0·91 (0·65-1·27) | 0·585 |
| Age2*Main occupation | | | | | | |
| Age2*Low skill | - | - | 0·99 (0·76-1·30) | 0·965 | 0·98 (0·84-1·13) | 0·761 |
| Age2*Never worked | - | - | 1·14 (0·66-1·95) | 0·642 | 1·11 (0·82-1·49) | 0·503 |
| Age*Financial strain | | | | | | |
| Age*Fairly easily | - | - | 1·01 (0·82-1·23) | 0·95 | 1·03 (0·90-1·18) | 0·666 |
| Age*Some difficulty | - | - | 1·08 (0·85-1·38) | 0·54 | 1·14 (0·97-1·34) | 0·103 |
| Age*Great difficulty | - | - | 0·79 (0·55-1·12) | 0·184 | 0·88 (0·69-1·11) | 0·265 |
| Age2*Financial strain | | | | | | |
| Age2*Fairly easily | - | - | 1·03 (0·85-1·25) | 0·759 | 0·99 (0·88-1·13) | 0·915 |
| Age2*Some difficulty | - | - | 1·00 (0·80-1·25) | 0·992 | 1·05 (0·91-1·21) | 0·498 |
| Age2*Great difficulty | - | - | 1·00 (0·73-1·36) | 0·988 | 0·98 (0·80-1·20) | 0·84 |
| Age*Obesity | - | - | - | - | 0·96 (0·81-1·13) | 0·596 |
| Age2*Obesity | - | - | - | - | 0·89 (0·78-1·03) | 0·108 |
| Age*High alcohol use | - | - | - | - | 0·93 (0·77-1·11) | 0·395 |
| Age2*High alcohol use | - | - | - | - | 1·00 (0·86-1·16) | 0·997 |
| Age*Low physical activity | - | - | - | - | 0·98 (0·87-1·11) | 0·761 |
| Age2*Low physical activity | - | - | - | - | 1·01 (0·90-1·13) | 0·913 |
| Age*Smoking | - | - | - | - | 0·96 (0·77-1·20) | 0·723 |
| Age2*Smoking | - | - | - | - | 1·13 (0·96-1·34) | 0·145 |
| Age*multimorbidity | - | - | - | - | 0·91 (0·81-1·02) | 0·118 |
| Age2*multimorbidity | - | - | - | - | 0·95 (0·86-1·06) | 0·352 |
| Age*depression | - | - | - | - | 0·99 (0·87-1·13) | 0·891 |
| Age2*depression | - | - | - | - | 1·04 (0·92-1·16) | 0·54 |
| Age*ADL | - | - | - | - | 0·88 (0·75-1·05) | 0·151 |
| Age2*ADL | - | - | - | - | 0·87 (0·74-1·01) | 0·074 |
| Age*Nursing home | - | - | - | - | 0·70 (0·14-3·43) | 0·663 |
| Age2*Nursing home | - | - | - | - | 0·46 (0·11-1·81) | 0·264 |
| *Legend*: OR=odds ratios; CI=confidence interval; All models were adjusted for birth cohorts, squared age, and countries. Model 3 additionally adjusted for obesity, alcohol consumption (at baseline), smoking (at baseline), physical activity, multimorbidity, depression, limitations in activities of daily living (ADL) and living situation (independent vs. nursing home). | | | | | | |

| **S6 Table.** Association of the life-course socioeconomic conditions (SECs) score with odds of polypharmacy at age 73 years and during ageing. (N = 21,794) | | | | |
| --- | --- | --- | --- | --- |
|  | **Model 1** |  | **Model 2** |  |
| **Variables** | OR (95% CI) | P-value | OR (95% CI) | P-value |
| *Linear age (10-year follow-up)* | 4·03 (2·66-6·09) | <0·001 | 2·18 (1·68-2·84) | <0·001 |
| *Squared age (10-year follow-up)* | 1·46 (1·08-1·97) | 0·015 | 1·06 (0·87-1·29) | 0·579 |
| *Sex (ref. women)* | 1·18 (0·97-1·44) | 0·089 | 1·40 (1·25-1·57) | <0·001 |
| *Life-course score* | 0·83 (0·77-0·89) | <0·001 | 0·87 (0·83-0·91) | <0·001 |
| *Obesity* | - | - | 2·70 (2·30-3·17) | <0·001 |
| *High alcohol consumption* | - | - | 1·01 (0·85-1·19) | 0·935 |
| *Low physicial activity* | - | - | 1·67 (1·45-1·92) | <0·001 |
| *Smoking* | - | - | 0·97 (0·82-1·16) | 0·766 |
| *Multimorbidity* | - | - | 5·68 (4·99-6·47) | <0·001 |
| *Depression* | - | - | 2·04 (1·76-2·36) | <0·001 |
| *ADL* | - | - | 2·88 (2·33-3·55) | <0·001 |
| Age*Life-course score | 1·00 (0·93-1·07) | 0·939 | 1·02 (0·99-1·06) | 0·215 |
| Age2*Llife-course score | 0·97 (0·91-1·03) | 0·311 | 1·00 (0·97-1·03) | 0·964 |
| Age*Obesity | - | - | 0·94 (0·81-1·11) | 0·475 |
| Age2*Obesity | - | - | 0·89 (0·78-1·02) | 0·081 |
| Age*High alcohol use | - | - | 1·01 (0·85-1·19) | 0·954 |
| Age2*High alcohol use | - | - | 1·00 (0·87-1·16) | 0·989 |
| Age*Low physical activity | - | - | 0·97 (0·86-1·09) | 0·577 |
| Age2*Low physical activity | - | - | 1·00 (0·90-1·12) | 0·992 |
| Age*Smoking | - | - | 0·93 (0·75-1·16) | 0·525 |
| Age2*Smoking | - | - | 1·13 (0·96-1·33) | 0·139 |
| Age*multimorbidity | - | - | 0·91 (0·81-1·02) | 0·094 |
| Age2*multimorbidity | - | - | 0·97 (0·87-1·07) | 0·529 |
| Age*depression | - | - | 0·99 (0·88-1·12) | 0·864 |
| Age2*depression | - | - | 1·00 (0·90-1·12) | 0·957 |
| Age*ADL | - | - | 0·89 (0·76-1·05) | 0·161 |
| Age2*ADL | - | - | 0·86 (0·74-1·01) | 0·06 |
| Age*Nursing home | - | - | 0·99 (0·20-4·86) | 0·991 |
| Age2*Nursing home | - | - | 0·33 (0·08-1·30) | 0·113 |
| $ |  |  |  |  |
| *Legend*: OR=odds ratios; CI=confidence interval. All models were adjusted for birth cohorts, squared age, and countries. Model 2 additionally adjusted for obesity, alcohol consumption (at baseline), smoking (at baseline), physical activity, multimorbidity, depression, limitations in activities of daily living (ADL) and living situation (independent vs. nursing home). | | | | |

| **S7 Table.**  Association of CSCs with odds of multimorbidity at age 73 years and rate of change in odds of multimorbidity during ageing, stratified by sex. (N = 31,775) | | | | | | | | | | | | |
| --- | --- | --- | --- | --- | --- | --- | --- | --- | --- | --- | --- | --- |
|  | **Women** |  |  |  |  |  | **Men** |  |  |  |  |  |
| Variables | OR (95% CI) | P-value | OR (95% CI) | P-value | OR (95% CI) | P-value | OR (95% CI) | P-value | OR (95% CI) | P-value | OR (95% CI) | P-value |
| **Risk** | **Model 1a** |  | **Model 2a** |  | **Model 3a** |  | **Model 1a** |  | **Model 2a** |  | **Model 3a** |  |
| *Age (10-year follow-up)* | 3·31 (2·89-3·80) | <0·001 | 3·38 (2·95-3·88) | <0·001 | 3·18 (2·78-3·64) | <0·001 | 3·53 (3·08-4·03) | <0·001 | 3·64 (3·18-4·17) | <0·001 | 3·49 (3·05-3·98) | <0·001 |
| *CSCs (ref. most disadvantaged)* | | | | | | | | | | | | |
| Disadvantaged | 0·64 (0·53-0·78) | <0·001 | 0·73 (0·61-0·89) | 0·001 | 0·75 (0·62-0·90) | 0·002 | 0·93 (0·79-1·11) | **0·432** | 1·02 (0·87-1·21) | **0·775** | 1·02 (0·87-1·20) | **0·814** |
| Middle | 0·44 (0·37-0·53) | <0·001 | 0·60 (0·49-0·72) | <0·001 | 0·62 (0·52-0·75) | <0·001 | 0·77 (0·65-0·91) | 0·003 | 0·95 (0·80-1·13) | **0·566** | 0·96 (0·81-1·13) | **0·634** |
| Advantaged | 0·39 (0·31-0·47) | <0·001 | 0·59 (0·47-0·72) | <0·001 | 0·63 (0·52-0·77) | <0·001 | 0·66 (0·55-0·80) | <0·001 | 0·89 (0·73-1·08) | **0·227** | 0·90 (0·75-1·08) | **0·253** |
| Most advantaged | 0·28 (0·22-0·36) | <0·001 | 0·51 (0·39-0·66) | <0·001 | 0·54 (0·42-0·70) | <0·001 | 0·56 (0·44-0·72) | <0·001 | 0·85 (0·66-1·09) | **0·199** | 0·87 (0·68-1·11) | **0·254** |
|  |  |  |  |  |  |  |  |  |  |  |  |  |
| **Risk with ageing** | **Model 1b** |  | **Model 2b** |  | **Model 3b** |  | **Model 1b** |  | **Model 2b** |  | **Model 3b** |  |
| *Age (10-year follow-up)* | 3·18 (2·59-3·91) | <0·001 | 3·03 (2·40-3·84) | <0·001 | 3·08 (2·35-4·04) | <0·001 | 3·43 (2·84-4·14) | <0·001 | 3·41 (2·73-4·27) | <0·001 | 3·48 (2·69-4·51) | <0·001 |
| *CSCs (ref. most disadvantaged)* | | | | | | | | | | | | |
| Disadvantaged | 0·63 (0·49-0·79) | <0·001 | 0·72 (0·56-0·91) | 0·006 | 0·74 (0·59-0·93) | 0·01 | 0·90 (0·73-1·11) | 0·338 | 0·97 (0·79-1·20) | 0·792 | 0·97 (0·79-1·19) | 0·768 |
| Middle | 0·42 (0·33-0·53) | <0·001 | 0·56 (0·44-0·71) | <0·001 | 0·60 (0·48-0·76) | <0·001 | 0·73 (0·59-0·89) | 0·002 | 0·89 (0·72-1·10) | 0·261 | 0·87 (0·71-1·07) | 0·195 |
| Advantaged | 0·39 (0·30-0·50) | <0·001 | 0·58 (0·44-0·75) | <0·001 | 0·63 (0·49-0·81) | <0·001 | 0·63 (0·50-0·80) | <0·001 | 0·83 (0·65-1·05) | 0·128 | 0·82 (0·65-1·03) | 0·092 |
| Most advantaged | 0·24 (0·17-0·33) | <0·001 | 0·40 (0·29-0·57) | <0·001 | 0·44 (0·32-0·62) | <0·001 | 0·64 (0·47-0·88) | 0·005 | 0·93 (0·67-1·29) | 0·661 | 0·94 (0·69-1·30) | 0·719 |
| Age*CSCs |  |  |  |  |  |  |  |  |  |  |  |  |
| Age*Disadvantaged | 1·00 (0·81-1·23) | 0·976 | 0·95 (0·77-1·17) | 0·630 | 0·97 (0·79-1·18) | 0·758 | 1·04 (0·86-1·26) | 0·659 | 1·02 (0·84-1·23) | 0·849 | 1·02 (0·85-1·23) | 0·804 |
| Age*Middle | 1·12 (0·91-1·38) | 0·279 | 1·00 (0·81-1·24) | 0·988 | 1·01 (0·82-1·24) | 0·908 | 1·06 (0·88-1·28) | 0·513 | 1·01 (0·83-1·22) | 0·940 | 1·00 (0·83-1·21) | 0·978 |
| Age*Advantaged | 1·16 (0·93-1·44) | 0·192 | 1·00 (0·79-1·26) | 0·992 | 1·00 (0·80-1·25) | 0·973 | 0·95 (0·77-1·18) | 0·660 | 0·88 (0·70-1·10) | 0·260 | 0·88 (0·71-1·10) | 0·257 |
| Age*Most advantaged | 0·80 (0·60-1·07) | 0·128 | 0·66 (0·49-0·90) | **0·008** | 0·68 (0·51-0·92) | **0·011** | 1·27 (0·97-1·65) | 0·080 | 1·07 (0·81-1·43) | 0·630 | 1·15 (0·87-1·51) | 0·332 |
| Age^2^*CSCs |  |  |  |  |  |  |  |  |  |  |  |  |
| Age^2^*Disadvantaged | 1·01 (0·85-1·21) | 0·904 | 1·00 (0·84-1·19) | 0·995 | 0·99 (0·83-1·18) | 0·909 | 1·04 (0·88-1·24) | 0·631 | 1·05 (0·89-1·25) | 0·567 | 1·06 (0·90-1·25) | 0·505 |
| Age^2^*Middle | 1·10 (0·92-1·30) | 0·293 | 1·07 (0·90-1·28) | 0·439 | 1·04 (0·88-1·24) | 0·629 | 1·08 (0·92-1·27) | 0·337 | 1·10 (0·93-1·31) | 0·255 | 1·11 (0·94-1·31) | 0·217 |
| Age^2^*Advantaged | 1·05 (0·88-1·26) | 0·564 | 1·03 (0·85-1·24) | 0·758 | 1·01 (0·84-1·21) | 0·929 | 1·01 (0·84-1·20) | 0·942 | 1·03 (0·85-1·24) | 0·793 | 1·03 (0·86-1·24) | 0·751 |
| Age^2^*Most advantaged | 1·00 (0·80-1·24) | 0·990 | 0·99 (0·78-1·24) | 0·900 | 0·98 (0·78-1·22) | 0·836 | 0·99 (0·80-1·23) | 0·911 | 1·02 (0·81-1·29) | 0·876 | 1·01 (0·81-1·27) | 0·917 |
| *Legend*: OR=odds ratios; CI=confidence interval. All models were adjusted for birth cohorts, attrition, squared age, and countries. Models 2 (a and b) additionally adjusted for education, main occupational position and financial strain. Models 3 (a and b) additionally adjusted for obesity, alcohol consumption, smoking and physical activity. Models 1b, 2b and 3b additionally adjusted for interactions terms of early SEC with age (1b), of education, main occupational position and financial strain with age (2b), and obesity, alcohol consumption, smoking and physical activity with age (3b). All models included random intercepts of participants. | | | | | | | | | | | | |

| **S8 Table** Association of the life-course SECs score with odds of multimorbidity at age 73 years and rate of change of odds of multimorbidity during ageing, stratified by sex. (N = 31,775) | | | | | | | | |
| --- | --- | --- | --- | --- | --- | --- | --- | --- |
| **Multimorbidity** |  |  |  |  |  |  |  |  |
|  | **Women** |  |  |  | **Men** |  |  |  |
| **Variables** | OR (95% CI) | P-value | OR (95% CI) | P-value | OR (95% CI) | P-value | OR (95% CI) | P-value |
| **Risk** | **Model 1a** |  | **Model 2a** |  | **Model 1a** |  | **Model 2a** |  |
| *Age (10-year follow-up)* | 3·28 (2·86-3·76) | <0·001 | 3·11 (2·72-3.56) | <0·001 | 3·55 (3·11-4·06) | <0·001 | 3·39 (2·97-3·87) | <0·001 |
| *Life-course score* | 0·71 (0·68-0·73) | <0·001 | 0.75 (0.72-0.77) | <0·001 | 0·78 (0·76-0·81) | <0·001 | 0·81 (0·79-0·84) | <0·001 |
|  |  |  |  |  |  |  |  |  |
| **Risk with ageing** | **Model 1b** |  | **Model 2b** |  | **Model 1b** |  | **Model 2b** |  |
| *Age (10-year follow-up)* | 2·89 (2·37-3·52) | <0·001 | 3·04 (2·37-3·90) | <0·001 | 3·15 (2·60-3·82) | <0·001 | 3·07 (2·54-3·72) | <0·001 |
| *Life-course score* | 0·72 (0·69-0·75) | <0·001 | 0·78 (0·75-0·81) | <0·001 | 0·81 (0·77-0·84) | <0·001 | 0·83 (0·80-0·86) | <0·001 |
| Age*Life-course score | 1·03 (0·99-1·07) | 0·105 | 1·02 (0·99-1·06) | 0·175 | 1·03 (1·00-1·06) | 0·089 | 1·03 (1·00-1·06) | 0·075 |
| Age^2^*Life-course score | 0·99 (0·97-1·02) | 0·678 | 1·00 (0·97-1·03) | 0·951 | 0·98 (0·96-1·01) | 0·228 | 0·99 (0·96-1·01) | 0·325 |
| *Legend*: OR=odds ratios; CI=confidence interval; SECs=socioeconomic circumstances. All models were adjusted for birth cohorts, attrition, squared age, and countries. Models 2 (a and b) additionally adjusted for obesity, alcohol consumption, smoking, physical activity for multimorbidity. Models 1b and 2b additionally adjusted for interactions terms of the score of life-course SEC with age (1b), and of obesity, alcohol consumption, smoking and physical activity with age (2b). All models included random intercepts of participants. Life-course SECs score: 0 (disadvantaged) to 8 (advantaged). | | | | | | | | |

| **S9 Table.** Association of CSCs with odds of polypharmacy at age 73 years and rate of change in odds of polypharmacy during ageing, stratified by sex. (N = 21,794) | | | | | | | | | | | | | |
| --- | --- | --- | --- | --- | --- | --- | --- | --- | --- | --- | --- | --- | --- |
|  | **Women** |  |  |  |  |  | **Men** |  |  |  |  |  |  |
|  | OR (95% CI) | P-value | OR (95% CI) | P-value | OR (95% CI) | P-value | OR (95% CI) | P-value | OR (95% CI) | P-value | OR (95% CI) | P-value |  |
| **Risk** | **Model 1a** |  | **Model 2a** |  | **Model 3a** |  | **Model 1a** |  | **Model 2a** |  | **Model 3a** |  |  |
| *Age (10-year follow-up)* | 3·38 (2·21-5·17) | <0·001 | 3·28 (2·15-5·02) | <0·001 | 2·16 (1·64-2·84) | <0·001 | 4·44 (2·43-8·11) | <0·001 | 3·25 (1·77-5·97) | <0·001 | 2·17 (1·70-2·77) | <0·001 |  |
| *CSCs (ref. most disadvantaged)* | | | | | | | | | | | | | |
| Disadvantaged | 0·77 (0·48-1·25) | 0·293 | 0·83 (0·51-1·35) | 0·446 | 0·90 (0·67-1·21) | 0·493 | 1·17 (0·36-3·79) | 0·792 | 1·35 (0·41-4·44) | 0·616 | 1·13 (0·89-1·43) | 0·326 |  |
| Middle | 0·60 (0·37-0·98) | 0·040 | 0·72 (0·44-1·19) | 0·199 | 0·82 (0·61-1·11) | 0·198 | 1·02 (0·31-3·35) | 0·968 | 0·99 (0·29-3·35) | 0·982 | 1·06 (0·83-1·36) | 0·621 |  |
| Advantaged | 0·53 (0·31-0·89) | 0·016 | 0·68 (0·39-1·17) | 0·163 | 0·83 (0·60-1·15) | 0·265 | 0·94 (0·25-3·53) | 0·924 | 1·04 (0·26-4·20) | 0·956 | 1·05 (0·80-1·38) | 0·715 |  |
| Most advantaged | 0·48 (0·24-0·94) | 0·032 | 0·65 (0·32-1·32) | 0·232 | 0·87 (0·57-1·32) | 0·505 | 0·88 (0·14-5·42) | 0·89 | 0·83 (0·12-5·70) | 0·851 | 0·99 (0·68-1·44) | 0·962 |  |
|  |  |  |  |  |  |  |  |  |  |  |  |  |  |
| **Risk with ageing** | **Model 1b** |  | **Model 2b** |  | **Model 3b** |  | **Model 1b** |  | **Model 2b** |  | **Model 3b** |  |  |
| *Age (10-year follow-up)* | 3·35 (1·81-6·19) | <0·001 | 3·27 (1·44-7·42) | 0·005 | 2·70 (1·57-4·62) | <0·001 | 4·34 (1·41-13·38) | 0·011 | 3·49 (0·75-16·22) | 0·11 | 2·27 (1·47-3·52) | <0·001 |  |
| *CSCs (ref. most disadvantaged)* | | | | | | | | | | | | | |
| Disadvantaged | 0·85 (0·47-1·52) | 0·58 | 1·01 (0·55-1·83) | 0·987 | 1·01 (0·69-1·49) | 0·956 | 1·12 (0·29-4·40) | 0·868 | 1·16 (0·30-4·54) | 0·829 | 1·12 (0·83-1·51) | 0·476 |  |
| Middle | 0·60 (0·34-1·07) | 0·086 | 0·85 (0·47-1·56) | 0·608 | 1·10 (0·75-1·61) | 0·628 | 0·94 (0·24-3·65) | 0·927 | 1·02 (0·26-4·06) | 0·976 | 1·25 (0·93-1·68) | 0·143 |  |
| Advantaged | 0·55 (0·30-1·04) | 0·066 | 0·78 (0·40-1·52) | 0·469 | 1·22 (0·80-1·85) | 0·363 | 1·00 (0·22-4·51) | 0·999 | 0·99 (0·21-4·78) | 0·991 | 1·33 (0·95-1·86) | 0·092 |  |
| Most advantaged | 0·50 (0·22-1·15) | 0·103 | 0·77 (0·32-1·84) | 0·549 | 1·15 (0·66-2·02) | 0·616 | 0·96 (0·12-7·82) | 0·969 | 1·06 (0·12-9·56) | 0·956 | 1·14 (0·72-1·82) | 0·572 |  |
| Age*CSCs |  |  |  |  |  |  |  |  |  |  |  |  |  |
| Age*Disadvantaged | 1·24 (0·69-2·22) | 0·476 | 1·22 (0·67-2·23) | 0·518 | 1·25 (0·87-1·78) | 0·224 | 0·86 (0·24-3·05) | 0·821 | 0·92 (0·26-3·27) | 0·891 | 0·92 (0·69-1·23) | 0·596 |  |
| Age*Middle | 1·05 (0·60-1·84) | 0·870 | 1·01 (0·55-1·84) | 0·981 | 1·05 (0·73-1·50) | 0·785 | 0·98 (0·30-3·29) | 0·980 | 0·95 (0·27-3·33) | 0·936 | 0·91 (0·68-1·22) | 0·538 |  |
| Age*Advantaged | 1·03 (0·57-1·86) | 0·935 | 0·87 (0·46-1·64) | 0·658 | 0·91 (0·61-1·34) | 0·624 | 1·04 (0·28-3·90) | 0·957 | 0·99 (0·24-4·01) | 0·989 | 1·01 (0·73-1·41) | 0·947 |  |
| Age*Most advantaged | 0·71 (0·34-1·49) | 0·367 | 0·62 (0·27-1·39) | 0·244 | 0·72 (0·43-1·18) | 0·190 | 0·85 (0·15-4·94) | 0·854 | 0·83 (0·13-5·36) | 0·847 | 0·78 (0·51-1·19) | 0·242 |  |
| Age^2^*CSCs |  |  |  |  |  |  |  |  |  |  |  |  |  |
| Age^2^*Disadvantaged | 0·92 (0·55-1·55) | 0·767 | 0·80 (0·48-1·35) | 0·408 | 0·94 (0·68-1·29) | 0·701 | 1·09 (0·40-2·97) | 0·862 | 1·00 (0·36-2·77) | 1·000 | 1·01 (0·77-1·33) | 0·935 |  |
| Age^2^*Middle | 1·00 (0·62-1·62) | 0·998 | 0·81 (0·49-1·34) | 0·411 | 0·95 (0·69-1·31) | 0·763 | 1·22 (0·48-3·11) | 0·677 | 1·12 (0·42-3·00) | 0·821 | 1·00 (0·76-1·31) | 0·995 |  |
| Age^2^*Advantaged | 0·94 (0·57-1·56) | 0·815 | 0·78 (0·46-1·33) | 0·358 | 0·85 (0·61-1·19) | 0·353 | 1·00 (0·36-2·79) | 0·999 | 1·00 (0·34-2·96) | 0·999 | 0·99 (0·73-1·35) | 0·972 |  |
| Age^2^*Most advantaged | 0·81 (0·44-1·49) | 0·499 | 0·71 (0·37-1·37) | 0·305 | 0·91 (0·60-1·37) | 0·645 | 0·92 (0·24-3·48) | 0·904 | 0·95 (0·23-3·89) | 0·946 | 0·95 (0·66-1·38) | 0·797 |  |
| *Legend*: OR=odds ratios; CI=confidence interval. All models were adjusted for birth cohorts, squared age, and countries. Models 2 (a and b) additionally adjusted for education, main occupational position and financial strain. Models 3 (a and b) additionally adjusted for obesity, alcohol consumption, smoking, physical activity, number of chronic conditions, depression, limitations in activities of daily living and living situation (independent vs. nursing home). Models 1b, 2b and 3b additionally adjusted for interactions terms of early SEC with age (1b), of education, main occupational position and financial strain with age (2b), and obesity, alcohol consumption, smoking and physical activity with age (3b). All models included random intercepts of participants. | | | | | | | | | | | | | |

| **S10 Table.** Association of life-course SECs score with odds of polypharmacy at age 73 years and rate of change in odds of polypharmacy during ageing, stratified by sex. (N = 21,794) | | | | | | | | |
| --- | --- | --- | --- | --- | --- | --- | --- | --- |
| **Polypharmacy** |  |  |  |  |  |  |  |  |
|  | **Women** |  |  |  | **Men** |  |  |  |
|  | OR (95% CI) | P-value | OR (95% CI) | P-value | OR (95% CI) | P-value | OR (95% CI) | P-value |
| **Risk** | **Model 1a** |  | **Model 2a** |  | **Model 1a** |  | **Model 2a** |  |
| *Age (10-year follow-up)* | 3·47 (2·27-5·31) | <0·001 | 2·12 (1·61-2·80) | <0·001 | 4·19 (2·29-7·64) | <0·001 | 2·20 (1·75-2·78) | <0·001 |
| *Life-course score* | 0·75 (0·69-0·83) | <0·001 | 0·81 (0·77-0·86) | <0·001 | 0·86 (0·68-1·09) | 0·208 | 0·92 (0·87-0·96) | <0·001 |
| **Risk with ageing** | **Model 1b** |  | **Model 2b** |  | **Model 1b** |  | **Model 2b** |  |
| *Age (10-year follow-up)* | 4·04 (2·22-7·32) | <0·001 | 2·45 (1·66-3·63) | <0·001 | 3·05 (1·05-8·90) | 0·041 | 1·88 (1·33-2·67) | <0·001 |
| *Life-course score* | 0·74 (0·67-0·83) | <0·001 | 0·82 (0·77-0·88) | <0·001 | 0·87 (0·67-1·14) | 0·315 | 0·93 (0·88-0·98) | 0·012 |
| Age***Life-course score | 0·96 (0·87-1·06) | 0·464 | 0·99 (0·94-1·04) | 0·657 | 1·04 (0·84-1·27) | 0·735 | 1·06 (1·01-1·11) | 0·028 |
| Age^2^*Life-course score | 1·01 (0·93-1·09) | 0·886 | 1·00 (0·95-1·04) | 0·823 | 0·98 (0·83-1·16) | 0·844 | 0·99 (0·95-1·04) | 0·771 |
| *Legend*: OR=odds ratios; CI=confidence interval; SECs=socioeconomic circumstances. All models were adjusted for birth cohorts, squared age, and countries. Models 2 (a and b) additionally adjusted for obesity, alcohol consumption, smoking, physical activity, number of chronic conditions, depression, limitations in activities of daily living and living situation (independent vs. nursing home). Models 1b and 2b additionally adjusted for interactions terms of the life-course score with age (1b), and obesity, alcohol consumption, smoking and physical activity with age, number of chronic conditions, depression, limitations in activities of daily living and living situation (independent vs. nursing home) (2b). All models included random intercepts of participants. Life-course SECs score: 0 (disadvantaged) to 8 (advantaged). | | | | | | | | |

| **S11 Table**. Baseline characteristics of study participants including information on countries included in the analysis. | | | | |
| --- | --- | --- | --- | --- |
|  | |  | **Cohort for multimorbidity analyses** | **Cohort for polypharmacy analyses** |
|  | |  | (N=31,432) | (N=21,794) |
| *Age, mean (SD)* | | | 66·2 (9·0) | 69·0 (8·9) |
| *Attrition* | | | | |
|  | No dropout | | 27,163 (86·4) | 21,527 (98·8) |
|  | Dropout | | 3,059 (9·7) | 0 (0) |
|  | Deceased | | 1,210 (3·9) | 267 (1·2) |
| *Sex* | | |  |  |
|  | Women | | 15,910 (50·6) | 11,075 (50·8) |
|  | Men | | 15,522 (49·4) | 10,719 (49·2) |
| *Birth cohort, n (%)* | | | | |
|  | 1919-1928 | | 1,331 (4·2) | 701 (3·2) |
|  | 1929-1938 | | 5,415 (17·3) | 3,978 (18·3) |
|  | 1939-1945 | | 6,923 (22·0) | 5,134 (23·6) |
|  | After 1945 | | 17,763 (56·5) | 11,981 (55·0) |
| *Polypharmacy, n (%)* | | | - | 5,318 (24·4) |
| *Multimorbidity^3^, n (%)* | | | 11,456 (36·5) | 9,616 (44·1) |
| *Childhood socioeconomic conditions, n (%)* | | | | |
|  | | Most disadvantaged | 4,061 (12·9) | 3,017 (13·8) |
|  | | Disadvantaged | 7,203 (22·9) | 5,163 (23·7) |
|  | | Middle | 10,786 (34·3) | 7,348 (33·7) |
|  | | Advantaged | 7,028 (22·4) | 4,732 (21·7) |
|  | | Most advantaged | 2,354 (7·5) | 1,534 (7·0) |
| *Education, n (%)* | | | | |
|  | | Primary | 5,941 (18·9) | 4,320 (19·8) |
|  | | Secondary | 18,011 (57·3) | 12,398 (56·9) |
|  | | Tertiary | 7,480 (23·8) | 5,076 (23·3) |
| *Main occupational position, n (%)* | | | | |
|  | | Low skilled | 10,917 (63·4) | 13,738 (63·0) |
|  | | High skilled | 10,244 (32·6) | 7,133 (32·7) |
|  | | Never worked | 1,271 (4·0) | 923 (4·2) |
| *Financial strain, n (%) (able to make ends meet)* | | | | |
|  | | Easily | 10,182 (32·4) | 8,038 (36·9) |
|  | | Fairly easily | 10,386 (33·0) | 6,226 (28·6) |
|  | | With some difficulty | 2,929 (9·3) | 2,059 (9·5) |
|  | | With great difficulty | 7,935 (25·3) | 5,471 (25·1) |
| *Obesity^1^, n (%)* | | | 6,152 (19·6) | 5,121 (23·5) |
| *Alcohol consumption^2^, n (%)* | | | | |
|  | | Ok | 23,758 (75·6) | 16,607 (76·2) |
|  | | Too much | 7,674 (24·4) | 5,187 (23·8) |
| *Smoking, n (%)* | | | 7,770 (24·7) | 5,235 (24·0) |
| *High physical activity (%)^6^* | | | 6,960 (22·1) | 5,596 (25·7) |
| *Limitation in activities of daily living^4^, n (%)* | | | - | 1,880 (8·6) |
| *Nursing home, n (%)* | | | | |
|  | | *Permanently or temporarily* | - | 30 (0·1) |
|  | | *Not living in nursing home* | - | 21,764 (99·9) |
| *Depression^5^, n (%)* | | | - | 5,349 (24·5) |
| *Countries* | | | | |
| Belgium | | | 3,263 (10.4) | 2,391 (11) |
| Austria | | | 2,233 (7.1) | 1,531 (7) |
| Denmark | | | 2,216 (7.1) | 1,424 (6.5) |
| France | | | 2,553 (8.1) | 1,567 (7.2) |
| Germany | | | 2,102 (6.7) | 1,434 (6.6) |
| Greece | | | 1,339 (4.3) | 1,109 (5.1) |
| Israel | | | - | 283 (1.3) |
| Italy | | | 2,389 (7.6) | 1,721 (7.9) |
| Netherlands | | | 1,253 (4.0) | - |
| Spain | | | 2,061 (6.6) | 1,569 (7.2) |
| Sweden | | | 2,232 (7.1) | 1,596 (7.3) |
| Switzerland | | | 2,023 (6.4) | 1,332 (6.1) |
| Czech Republic | | | 2,669 (8.5) | 2,090 (9.6) |
| Poland | | | 1,057 (3.4) | 601 (2.8) |
| Estonia | | | 2,197 (7.0) | 1,669 (7.7) |
| Portugal | | | 87 (0.3) | 71 (0.3) |
| Slovenia | | | 1,149 (3.7) | 928 (4.3) |
| Luxembourg | | | 278 (0.9) | 227 (1) |
| Croatia | | | 331 (1.1) | 251 (1.2) |
| *Legend*: ^1^ BMI ≥30 kg/m^2^; ^2^ self-reported; ^3^ ≥2 chronic conditions; ^4^ ≥1 on activities of daily living scale; ^5^ ≥4 on EURO-Depression scale; ^6^ As used in Boris et al., 2018, Med Sci Sports Exerc, 2 variables were used to assess the level of daily life physical activity. The first item assessed vigorous physical activity (“How often do you engage in vigorous physical activity, such as sports, heavy housework, or a job that involves physical labour?”). The second item assessed moderate physical activity (“How often do you engage in activities that require a low or moderate level of energy such as gardening, cleaning the car, or doing a walk?”). Participants answered by using a 4-point scale (1, >1 a week; 2, once a week; 3, 1-3 times a month; 4, hardly ever, or never). Participants who did not answer “1” to either question were classified as “physically inactive”. | | | | |

| **S12 Table.** Association of childhood socioeconomic conditions (CSCs) with odds of multimorbidity at age 73 years, including country coefficients (N = 31,432) | | | | | | |
| --- | --- | --- | --- | --- | --- | --- |
|  | **Model 1** |  | **Model 2** |  | **Model 3** |  |
| *Variables* | *OR (95% CI)* | *P-value* | *OR (95% CI)* | *P-value* | *OR (95% CI)* | *P-value* |
| Linear age (10-year follow-up) | 3·47 (3·15-3·82) | <0·001 | 3·53 (3·21-3·89) | <0·001 | 3·36 (3·06-3·69) | <0·001 |
| Squared age (10-year follow-up) | 0·89 (0·85-0·93) | <0·001 | 0·88 (0·84-0·92) | <0·001 | 0·89 (0·85-0·93) | <0·001 |
| *Sex (ref. women)* | 0·83 (0·78-0·90) | <0·001 | 0·88 (0·82-0·95) | 0·001 | 0·87 (0·81-0·93) | <0·001 |
| *CSCs (ref. most disadvantaged)* | | | | | | |
| Disadvantaged | 0·79 (0·70-0·90) | <0·001 | 0·89 (0·78-1·01) | 0·061 | 0·89 (0·79-1·01) | 0·066 |
| Middle | 0·60 (0·53-0·68) | <0·001 | 0·77 (0·68-0·87) | <0·001 | 0·79 (0·70-0·90) | <0·001 |
| Advantaged | 0·52 (0·45-0·60) | <0·001 | 0·74 (0·64-0·85) | <0·001 | 0·77 (0·68-0·89) | <0·001 |
| Most advantaged | 0·40 (0·34-0·48) | <0·001 | 0·66 (0·55-0·80) | <0·001 | 0·70 (0·58-0·83) | <0·001 |
| *Education (ref. primary)* | | | | | | |
| Secondary | - | - | 0·67 (0·60-0·74) | <0·001 | 0·72 (0·65-0·80) | <0·001 |
| Tertiary | - | - | 0·47 (0·41-0·54) | <0·001 | 0·54 (0·47-0·62) | <0·001 |
| *Main occupation (ref. high skill)* | | | | | | |
| Low skill | - | - | 0·86 (0·78-0·94) | 0·001 | 0·89 (0·82-0·97) | 0·006 |
| Never worked | - | - | 0·98 (0·81-1·18) | 0·809 | 0·98 (0·81-1·18) | 0·814 |
| *Financial strain (able to make ends meet) (ref. easily)* | | | | | | |
| Fairly easily | - | - | 1·13 (1·06-1·20) | <0·001 | 1·10 (1·04-1·17) | 0·002 |
| Some difficulty | - | - | 1·48 (1·37-1·60) | <0·001 | 1·42 (1·31-1·53) | <0·001 |
| Great difficulty | - | - | 2·05 (1·83-2·29) | <0·001 | 1·95 (1·74-2·18) | <0·001 |
| *Countries (ref. Belgium)* | | | | | | |
| Belgium (reference) |  |  |  |  |  |  |
| Austria | 0·48 (0·41-0·57) | <0·001 | 0·51 (0·43-0·61) | <0·001 | 0·51 (0·44-0·61) | <0·001 |
| Denmark | 0·60 (0·51-0·72) | <0·001 | 0·67 (0·57-0·80) | <0·001 | 0·71 (0·61-0·84) | <0·001 |
| France | 0·78 (0·66-0·92) | 0·003 | 0·73 (0·62-0·86) | <0·001 | 0·74 (0·63-0·87) | <0·001 |
| Germany | 0·88 (0·74-1·05) | 0·157 | 0·95 (0·80-1·13) | 0·562 | 0·93 (0·79-1·10) | 0·4 |
| Greece | 0·77 (0·61-0·98) | 0·036 | 0·55 (0·43-0·70) | <0·001 | 0·57 (0·45-0·71) | <0·001 |
| Israel | 0·58 (0·40-0·85) | 0·005 | 0·57 (0·39-0·83) | 0·003 | 0·59 (0·41-0·85) | 0·004 |
| Italy | 0·52 (0·43-0·61) | <0·001 | 0·39 (0·33-0·47) | <0·001 | 0·43 (0·36-0·50) | <0·001 |
| Netherlands | 0·26 (0·20-0·35) | <0·001 | 0·30 (0·23-0·39) | <0·001 | 0·32 (0·25-0·42) | <0·001 |
| Spain | 0·74 (0·62-0·89) | 0·001 | 0·57 (0·47-0·68) | <0·001 | 0·60 (0·51-0·72) | <0·001 |
| Sweden | 0·29 (0·24-0·35) | <0·001 | 0·30 (0·25-0·36) | <0·001 | 0·33 (0·28-0·39) | <0·001 |
| Switzerland | 0·21 (0·17-0·25) | <0·001 | 0·21 (0·17-0·25) | <0·001 | 0·23 (0·19-0·28) | <0·001 |
| Czech Republic | 1·24 (1·05-1·45) | 0·011 | 1·10 (0·93-1·29) | 0·265 | 0·98 (0·84-1·15) | 0·796 |
| Poland | 0·82 (0·65-1·05) | 0·114 | 0·66 (0·52-0·84) | 0·001 | 0·63 (0·50-0·80) | <0·001 |
| Estonia | 0·60 (0·50-0·71) | <0·001 | 0·59 (0·49-0·70) | <0·001 | 0·54 (0·46-0·64) | <0·001 |
| Portugal | 1·16 (0·51-2·64) | 0·732 | 0·85 (0·38-1·94) | 0·706 | 0·98 (0·44-2·15) | 0·953 |
| Slovenia | 0·50 (0·40-0·62) | <0·001 | 0·46 (0·37-0·58) | <0·001 | 0·47 (0·38-0·59) | <0·001 |
| Luxembourg | 1·85 (1·21-2·83) | 0·004 | 1·66 (1·09-2·53) | 0·017 | 1·53 (1·02-2·29) | 0·038 |
| Croatia | 0·90 (0·58-1·40) | 0·645 | 0·69 (0·45-1·07) | 0·098 | 0·68 (0·45-1·04) | 0·076 |
| *Legend*: OR=odds ratios; CI=confidence interval. All models were adjusted for birth cohorts, attrition, and countries. Model 3 additionally adjusted for obesity, alcohol consumption (at baseline), smoking (at baseline) and physical activity. | | | | | | |

| **S13 Table.** Association of life-course socioeconomic circumstances (SECs) score with odds of multimorbidity at age 73 years, including country coefficients (N = 31,432) | | | | |
| --- | --- | --- | --- | --- |
|  | **Model 1** |  | **Model 2** |  |
| *Variables* | *OR (95% CI)* | *P-value* | *OR (95% CI)* | *P-value* |
| Linear age (10-year follow-up) | 3·44 (3·12-3·78) | <0·001 | 3·28 (2·99-3·61) | <0·001 |
| Squared age (10-year follow-up) | 0·88 (0·84-0·92) | <0·001 | 0·89 (0·85-0·93) | <0·001 |
| Sex (ref. women) | 0·91 (0·85-0·98) | 0·01 | 0·89 (0·83-0·96) | 0·001 |
| Life-course score (per-unit increase)^a^ | 0·74 (0·73-0·76) | <0·001 | 0·78 (0·76-0·80) | <0·001 |
| *Countries (ref. Belgium)* | | | | |
| Austria | 0·51 (0·43-0·60) | <0·001 | 0·51 (0·43-0·60) | <0·001 |
| Denmark | 0·72 (0·60-0·85) | <0·001 | 0·75 (0·64-0·89) | 0·001 |
| France | 0·72 (0·61-0·85) | <0·001 | 0·74 (0·63-0·86) | <0·001 |
| Germany | 0·96 (0·81-1·15) | 0·672 | 0·92 (0·77-1·08) | 0·309 |
| Greece | 0·52 (0·41-0·66) | <0·001 | 0·55 (0·44-0·70) | <0·001 |
| Israel | 0·58 (0·40-0·85) | 0·005 | 0·61 (0·42-0·87) | 0·007 |
| Italy | 0·36 (0·30-0·43) | <0·001 | 0·40 (0·34-0·47) | <0·001 |
| Netherlands | 0·31 (0·23-0·41) | <0·001 | 0·33 (0·25-0·43) | <0·001 |
| Spain | 0·55 (0·46-0·65) | <0·001 | 0·59 (0·49-0·70) | <0·001 |
| Sweden | 0·32 (0·27-0·38) | <0·001 | 0·35 (0·29-0·41) | <0·001 |
| Switzerland | 0·22 (0·18-0·26) | <0·001 | 0·24 (0·20-0·28) | <0·001 |
| Czech Republic | 1·09 (0·93-1·28) | 0·302 | 0·98 (0·84-1·15) | 0·83 |
| Poland | 0·62 (0·48-0·78) | <0·001 | 0·61 (0·48-0·76) | <0·001 |
| Estonia | 0·56 (0·47-0·66) | <0·001 | 0·52 (0·44-0·62) | <0·001 |
| Portugal | 0·78 (0·34-1·78) | 0·552 | 0·99 (0·45-2·18) | 0·973 |
| Slovenia | 0·43 (0·35-0·54) | <0·001 | 0·45 (0·36-0·56) | <0·001 |
| Luxembourg | 1·69 (1·11-2·58) | 0·014 | 1·57 (1·05-2·35) | 0·029 |
| Croatia | 0·66 (0·43-1·03) | 0·066 | 0·69 (0·45-1·05) | 0·085 |
|  |  |  |  |  |
| *Legend*: OR=odds ratios; CI=confidence interval. All models were adjusted for birth cohorts, attrition and countries. Model 2 additionally adjusted for obesity, alcohol consumption (at baseline), smoking (at baseline) and physical activity.  ^a^ Range of the life-course score: 0 to 8. A higher life-course score means a longer life-time exposure to advantaged SECs during the entire life course. | | | | |

| **S14 Table.** Association of childhood socioeconomic conditions (CSCs) with odds of polypharmacy at age 73 years, including country coefficients (N = 21,794) | | | | | | |
| --- | --- | --- | --- | --- | --- | --- |
|  | **Model 1** |  | **Model 2** |  | **Model 3** |  |
| **Variables** | *OR (95% CI)* | *P-value* | *OR (95% CI)* | *P-value* | *OR (95% CI)* | *P-value* |
| Linear age (10-year follow-up) | 4·09 (3·03-5·51) | <0·001 | 4·15 (3·08-5·60) | <0·001 | 2·14 (1·78-2·57) | <0·001 |
| Squared age (10-year follow-up) | 1·29 (1·11-1·50) | 0·001 | 1·28 (1·10-1·49) | 0·001 | 1·00 (0·91-1·09) | 0·946 |
| *Sex (ref. women)* | 1·12 (0·92-1·36) | 0·259 | 1·18 (0·96-1·43) | 0·112 | 1·40 (1·25-1·58) | <0·001 |
| *CSCs (ref. most disadvantaged)* | | | | | | |
| Disadvantaged | 0·97 (0·70-1·34) | 0·835 | 0·99 (0·71-1·38) | 0·961 | 1·01 (0·84-1·22) | 0·900 |
| Middle | 0·81 (0·58-1·12) | 0·205 | 0·91 (0·65-1·28) | 0·592 | 0·98 (0·81-1·18) | 0·799 |
| Advantaged | 0·73 (0·51-1·04) | 0·082 | 0·86 (0·59-1·25) | 0·429 | 0·97 (0·79-1·20) | 0·793 |
| Most advantaged | 0·68 (0·42-1·09) | 0·111 | 0·85 (0·51-1·40) | 0·512 | 0·91 (0·69-1·21) | 0·529 |
| *Education (ref. primary)* | | | | | | |
| Secondary | - | - | 0·75 (0·56-1·00) | 0·047 | 0·80 (0·68-0·94) | 0·006 |
| Tertiary | - | - | 0·66 (0·46-0·96) | 0·03 | 0·76 (0·62-0·93) | 0·009 |
| *Main occupation (ref. high skill)* | | | | | | |
| Low skill | - | - | 1·08 (0·85-1·38) | 0·506 | 1·03 (0·90-1·18) | 0·673 |
| Never worked | - | - | 1·22 (0·70-2·13) | 0·477 | 1·10 (0·81-1·50) | 0·548 |
| *Financial strain (able to make ends meet) (ref. easily)* | | | | | | |
| Fairly easily | - | - | 1·19 (1·00-1·42) | 0·054 | 1·17 (1·04-1·31) | 0·008 |
| Some difficulty | - | - | 1·37 (1·11-·1·69) | 0·003 | 1·34 (1·17-1·53) | <0·001 |
| Great difficulty | - | - | 1·93 (1·42-2·63) | <0·001 | 1·74 (1·43-2·11) | <0·001 |
| *Countries (ref. Belgium)* | | | | | | |
| Austria | 0·67 (0·42-1·06) | 0·087 | 0·68 (0·43-1·08) | 0·105 | 0·79 (0·61-1·02) | 0·068 |
| Croatia | 0·71 (0·26-1·98) | 0·515 | 0·55 (0·20-1·55) | 0·261 | 0·34 (0·18-0·63) | 0·001 |
| Czech Republic | 1·70 (1·12-2·56) | 0·012 | 1·59 (1·05-2·40) | 0·029 | 1·64 (1·29-2·07) | <0·001 |
| Denmark | 1·29 (0·81-2·05) | 0·276 | 1·39 (0·87-2·21) | 0·167 | 1·75 (1·35-2·28) | <0·001 |
| Estonia | 0·52 (0·33-0·83) | 0·006 | 0·48 (0·30-0·77) | 0·002 | 0·36 (0·28-0·47) | <0·001 |
| France | 0·82 (0·52-1·29) | 0·383 | 0·75 (0·48-1·19) | 0·224 | 0·66 (0·51-0·86) | 0·002 |
| Germany | 0·80 (0·50-1·27) | 0·338 | 0·82 (0·52-1·31) | 0·415 | 0·71 (0·55-0·92) | 0·011 |
| Greece | 0·35 (0·19-0·64) | 0·001 | 0·23 (0·13-0·44) | <0·001 | 0·12 (0·09-0·18) | <0·001 |
| Israel | 1·75 (0·76-4·06) | 0·191 | 1·63 (0·70-3·80) | 0·26 | 1·68 (1·02-2·74) | 0·04 |
| Italy | 0·59 (0·37-0·93) | 0·023 | 0·45 (0·28-0·72) | 0·001 | 0·45 (0·35-0·59) | <0·001 |
| Luxembourg | 0·91 (0·34-2·45) | 0·853 | 0·83 (0·31-2·24) | 0·709 | 0·57 (0·32-1·01) | 0·053 |
| Poland | 2·95 (1·57-5·54) | 0·001 | 2·47 (1·31-4·68) | 0·005 | 1·90 (1·29-2·80) | 0·001 |
| Portugal | 1·56 (0·31-7·98) | 0·591 | 0·93 (0·17-5·03) | 0·931 | 1·20 (0·44-3·32) | 0·722 |
| Slovenia | 0·41 (0·23-0·73) | 0·003 | 0·37 (0·20-0·67) | 0·001 | 0·29 (0·21-0·41) | <0·001 |
| Spain | 0·59 (0·37-0·95) | 0·029 | 0·46 (0·29-0·75) | 0·002 | 0·46 (0·35-0·61) | <0·001 |
| Sweden | 0·87 (0·56-1·36) | 0·555 | 0·88 (0·56-1·37) | 0·563 | 1·27 (0·99-1·64) | 0·064 |
| Switzerland | 0·45 (0·27-0·74) | 0·002 | 0·45 (0·27-0·75) | 0·002 | 0·59 (0·45-0·78) | <0·001 |
|  |  |  |  |  |  |  |
| *Legend*: OR=odds ratios; CI=confidence interval; All models were adjusted for birth cohorts, squared age, and countries. Model 3 additionally adjusted for obesity, alcohol consumption (at baseline), smoking (at baseline), physical activity, multimorbidity, depression, limitation with activities of daily activity and living in a nursing home. | | | | | | |

| **S15 Table.** Association of life-course SECs score with odds of polypharmacy at age 73 years, including country coefficients (N = 21,794) | | | | |
| --- | --- | --- | --- | --- |
|  | **Model 1** |  | **Model 2** |  |
| *Variables* | *OR (95% CI)* | *P-value* | *OR (95% CI)* | *P-value* |
| Linear age (10-year follow-up) | 2·84 (1·85-4·35) | <0·001 | 2·10 (1·75-2·52) | <0·001 |
| Squared age (10-year follow-up) | 1·00 (0·79-1·26) | 0·997 | 1·00 (0·92-1·09) | 0·982 |
| Sex (ref. women) | 1·16 (0·69-1·96) | 0·578 | 1·41 (1·26-1·58) | <0·001 |
| Life-course score (per-unit increase) ^a^ | 0·81 (0·69-0·96) | 0·012 | 0·86 (0·83-0·90) | <0·001 |
| *Countries (ref. Belgium)* | | | | |
| Austria | 0·66 (0·19-2·25) | 0·507 | 0·74 (0·57-0·96) | 0·023 |
| Croatia | 0·52 (0·02-1·51) | 0·7 | 0·33 (0·18-0·62) | <0·001 |
| Czech Republic | 1·41 (0·50-4·00) | 0·514 | 1·59 (1·26-2·00) | <0·001 |
| Denmark | 1·42 (0·44-4·64) | 0·557 | 1·77 (1·37-2·30) | <0·001 |
| Estonia | 0·44 (0·13-1·53) | 0·197 | 0·36 (0·27-0·46) | <0·001 |
| France | 0·68 (0·20-2·28) | 0·534 | 0·64 (0·50-0·83) | 0·001 |
| Germany | 0·76 (0·21-2·71) | 0·673 | 0·70 (0·54-0·91) | 0·008 |
| Greece | 0·23 (0·03-1·55) | 0·131 | 0·13 (0·09-0·18) | <0·001 |
| Israel | 1·26 (0·15-10·89) | 0·832 | 1·81 (1·11-2·97) | 0·018 |
| Italy | 0·39 (0·11-1·35) | 0·138 | 0·43 (0·33-0·55) | <0·001 |
| Luxembourg | 0·83 (0·04-15·42) | 0·9 | 0·60 (0·33-1·06) | 0·077 |
| Poland | 2·27 (0·47-10·98) | 0·306 | 1·68 (1·14-2·48) | 0·009 |
| Portugal | 0·99 (0·01-95·57) | 0·998 | 1·34 (0·48-3·69) | 0·578 |
| Slovenia | 0·33 (0·06-1·76) | 0·194 | 0·27 (0·20-0·38) | <0·001 |
| Spain | 0·42 (0·12-1·52) | 0·189 | 0·44 (0·34-0·58) | <0·001 |
| Sweden | 0·74 (0·23-2·40) | 0·622 | 1·30 (1·01-1·67) | 0·041 |
| Switzerland | 0·44 (0·11-1·75) | 0·243 | 0·58 (0·44-0·77) | <0·001 |
|  |  |  |  |  |
| *Legend*: OR=odds ratios; CI=confidence interval; SEC=socioeconomic conditions. All models were adjusted for birth cohorts, squared age, and countries. Model 2 additionally adjusted for obesity, alcohol consumption (at baseline), smoking (at baseline), physical activity, multimorbidity, depression, limitation with activities of daily activity and living in a nursing home.  ^a^ Range of the life-course score: 0-8, with 0 indicating a disadvantaged life course and 8 an advantaged life course. A higher score means longer life-time exposure to advantaged SECs during the entire life course | | | | |

**S16 Table: Results of sensitivity analyses**

|  | **Description** | **Rationale and main results** |
| --- | --- | --- |
| 1 | Exclude participants who died during follow-up | *Rationale:* to check if results would change when increasing the health selection bias (exclusion of participants who died during follow-up)  *Results:*  Multimorbidity: Models 1 to 3 provided similar results as the main analysis. When adding age interactions with childhood socioeconomic conditions (CSCs) and adulthood socioeconomic conditions (ASCs) predictors, results were similar too. *Life-course score*: Models 1 to 2 with the score of life-course SEC provided similar results as the main analysis. When adding age interactions with the score of life-course socioeconomic circumstances (hereafter life-course score), results were similar too. Of note, the statistical significance of the interaction between squared age and the score of life-course SEC was borderline (OR=0·98 95%CI 0·96-1·00, p=0·048) in model 1, but was not associated in model 2 (OR=0·99 95%CI 0·97-1·01).  Polypharmacy: Contrary to the main analysis, in model 1, CSC were associated with polypharmacy, with a gradient across estimates of the CSC categories: disadvantaged CSC OR=0·44, 95% CI 0·32-0·62; middle CSC OR=0·43, 95% CI 0·31-0·60; advantaged CSC OR=0·38, 95% CI 0·27-0·55; most advantaged CSC OR=0·37, 95% CI 0·23-0·59. When adjusting with ASC (model 2), all CSC categories were no longer associated (disadvantaged CSC OR=1·00, 95% CI 0·72-1·39; middle CSC OR=0·93, 95% CI 0·67-1·31; advantaged CSC OR=0·90, 95% CI 0·62-1·31; most advantaged CSC OR=0·88, 95% CI 0·53-1·45). When adding polypharmacy risk factors (model 3), all CSC estimates were even closer to 1.00. When adding age interactions with CSC and ASC predictors, results were similar to those of the main analysis. *Life-course score*: Models 1 to 2 with the score of life-course SEC provided similar results. When adding age interactions with the life-course score, results remained similar. |
| 2 | Exclude participants who dropped out during follow-up | *Rationale:* to check if results would change when increasing the health selection bias (exclusion of participants who drop-out during the follow-up)  *Results:*  Multimorbidity: Models 1 to 3 provided similar results as the main analysis. When adding age interactions with CSC and ASC predictors, results were similar too. *Life-course score*: Models 1 to 2 with the score of life-course SEC provided similar results as the main analysis. When adding age interactions with the life-course score, results were similar too. Of note, the statistical significance of the interaction between linear age and the score of life-course SEC was borderline (OR=1·03 95%CI 1·00-1·05, p=0·044) in model 1, but was not associated in model 2 (OR=1·02 95%CI 0·99-1·04).  Polypharmacy: not applicable because polypharmacy was only assessed once (during the last two waves of data collection) |
| 3 | Exclude participants aged 90 years and above at baseline | *Rationale:* to check if results would change when increasing the health selection bias (exclusion of very old participants)  *Results:*  Multimorbidity: Models 1 to 3 provided similar results as the main analysis. When adding age interactions with CSC and ASC predictors, results were similar too. *Life-course score*: Models 1 to 2 with the score of life-course SEC provided similar results with main analysis. When adding age interactions with the life-course score, results were similar too. Of note, the statistical significance of the interaction between linear age and the score of life-course SEC was borderline (OR=1·03 95%CI 1·00-1·05, p=0·05) in model 1, but was not associated in model 2 (OR=1·02 95%CI 0·99-1·04).  Polypharmacy: Models 1 to 3 provided similar results with main analysis. When adding age interactions with CSC and ASC predictors, results were similar to the ones of the main analysis. *Life-course score*: Models 1 to 2 with the score of life-course SEC provided similar results. When adding age interactions with the life-course score, results were similar too. |
| 4 | Included two indicators of cognitive performance, memory and verbal fluency, in the covariates. | *Rationale:* to check if results would change when adjusting with a known predictor of multimorbidity  *Results:*  Multimorbidity: Model 3 provided similar results as the main analysis. When adding age interactions with CSC and ASC predictors, results remained similar. *Life-course score*: Model 2 with the score of life-course SEC provided similar results as the main analysis. When adding age interactions with the life-course score, results were similar too. Of note, the interaction between linear age and the life-course score was statistically significant but borderline (OR=1·03 95%CI 1·00-1·05, p=0·025) in model 1, but was not associated in model 2 (OR=1·01 95%CI 0·98-1·03).  Polypharmacy: Models 3 provided similar results with main analysis. When adding age interactions with CSC and ASC predictors, results were similar with main analysis. *Life-course score*: Model 2 with the score of life-course SEC provided similar results. When adding age interactions with the life-course score, results were similar too. |

**S17 Table: Results of robustness analyses**

|  | **Description** | **Rationale and main results** |
| --- | --- | --- |
| 1 | Treating multimorbidity as a continuous score | Rationale: to check if results would change when treating multimorbidity as a continuous sum score of chronic conditions.  Results: Using multimorbidity as a continuous variable, models 1 to 3 provided similar results compared main analysis (i.e., treating multimorbidity as a dichotomic variable). When adding age interactions with CSC and ASC predictors, results were similar too (no signification interactions). Life-course score: Models 1 to 2 with the life-course score provided similar results with main analysis. When adding age interactions with the life-course score, results were similar too (no signification interactions). |
| 2 | Including the category “Other” in the score | Rationale: to check if results would change when including the other category in the sum score of chronic conditions (like in main analysis, multimorbidity was defined as ≥2 chronic conditions and assessed with multilevel logistic regressions).  Results: Models 1 to 3 provided similar results compared main analysis. One minor exception: while in main analysis disadvantaged CSCs became not significant in models 2 and 3, here disadvantaged CSC remained significantly associated across the three models (M1: OR=0·79, 95% CI 0·70-0·89; M2: OR=0·87, 95% CI 0·77-0·98; M3: OR=0·88, 95% CI 0·78-0·99). When adding age interactions with CSC and ASC predictors, results were similar too (no signification interactions). One exception: in Model 1, a significant interaction between linear age and middle CSC was observed (OR=1·15, 95% CI 1·01-1·31). This interaction remained not significant when adjusting for ASC (Model 2) and risk factors (M3). Life-course score: Models 1 to 2 with the score of life-course SEC provided similar results with main analysis. When adding age interactions with the life-course score, results were not similar to main analysis: the interaction of the life-course score with linear age was significant in model 1 (OR=1·04, 95% CI 1·02-1·06) and model 2 (OR=1·03, 95% CI 1·01-1·06). Squared age interactions were not significant. This result suggested that the linear evolution (intra-individual ageing) in the risk of multimorbidity was quicker as the longer life-time exposure to advantaged SECs increased, suggesting an adjustment phenomenon: as participants with longer life-time exposure of advantaged SECs have lower multimorbidity levels compared to participants without life-time exposure to socioeconomic advantages, during aging, they have a higher rate of increase of multimorbidity, catching up with multimorbidity levels of the disadvantaged. |
| 3 | Because information about asthma, arthritis, osteoporosis was only collected in waves 1, 2 and 4, and benign tumours in waves 2 and 4, we imputed the last recorded value to the following waves. The imputed score took the last available value for each item and copied it for the subsequent waves (waves 5, 6 and 7). | Rationale: to check if results would change when imputing information for asthma, arthritis, osteoporosis, and benign tumours in waves 5, 6 and 7 (like in main analysis, multimorbidity was defined as ≥2 chronic conditions and assessed with multilevel logistic regressions)  Results: Models 1 to 3 provided similar results compared main analysis. When adding age interactions with CSC and ASC predictors, results were similar too (no signification interactions). Life-course score: Models 1 to 2 with the life-course score provided similar results with main analysis. When adding age interactions with the life-course score, results were not similar to main analysis: the interaction of the life-course score with linear age was significant in model 1 (OR=1·03, 95% CI 1·01-1·06) but not in model 2 (OR=1·03, 95% CI 1·00-1·05, p-value=0.068). This result suggested that the linear evolution (intra-individual ageing) in the risk of multimorbidity was quicker as the longer life-time exposure to advantaged SECs increased. However, this effect was not robust to risk factors adjustment (model 2). |

**S18 Appendix.** Causality implications

We performed an associations study and did not use a causal inference approach. Therefore, we cannot speak of a causal effect of life-course factors on our study outcomes. Nevertheless, we emphasize a couple of facts that point in the direction of a causal effect, which needs to be further explored with additional research. First, in our analyses, we ensured that the exposures were collected before the outcomes. Although this is evident for the CSCs, we introduced a time lag to make sure that the information on ASCs and risk factors was collected before the information on study outcomes. Second, with the social gradient we observed in our results with CSCs, we saw a dose–response relation: The more advantaged the life-course factors of the respondents, the lower the odds of multimorbidity. Third, CSCs was previously found as a good predictor of many other health outcomes (physical, cognitive and emotional health) in old age, even in the context of ASC adjustment.[1] Fourth, the biological plausibility of the life-course factors and our study outcomes was not tested in the present study and remains highly hypothetical. The uncertainty is all the greater because many biological mechanisms can operate between life-course socioeconomic disadvantages and our outcomes, including epigenetics mechanism[2,3] inflammatory mechanisms,[4-6] and microbiome mechanisms.[7-10]

References

1. Cheval B, Orsholits D, Sieber S, Stringhini S, Courvoisier D, Kliegel M, et al. Early-life socioeconomic circumstances explain health differences in old age, but not their evolution over time. Journal of Epidemiology and Community Health. 2019;73(8):703-11.

2. Hughes A, Smart M, Gorrie-Stone T, Hannon E, Mill J, Bao Y, et al. Socioeconomic Position and DNA Methylation Age Acceleration Across the Life Course. American journal of epidemiology. 2018;187(11):2346-54.

3. Fiorito G, Polidoro S, Dugué P-A, Kivimaki M, Ponzi E, Matullo G, et al. Social adversity and epigenetic aging: a multi-cohort study on socioeconomic differences in peripheral blood DNA methylation. Scientific Reports. 2017;7(1):16266.

4. Castagné R, Kelly-Irving M, Campanella G, Guida F, Krogh V, Palli D, et al. Biological marks of early-life socioeconomic experience is detected in the adult inflammatory transcriptome. Scientific Reports. 2016;6(1):38705.

5. Castagné R, Delpierre C, Kelly-Irving M, Campanella G, Guida F, Krogh V, et al. A life course approach to explore the biological embedding of socioeconomic position and social mobility through circulating inflammatory markers. Scientific Reports. 2016;6(1):25170.

6. Pollitt RA, Kaufman JS, Rose KM, Diez-Roux AV, Zeng D, Heiss G. Cumulative life course and adult socioeconomic status and markers of inflammation in adulthood. Journal of Epidemiology and Community Health. 2008;62(6):484-91.

7. Wirbel J, Pyl PT, Kartal E, Zych K, Kashani A, Milanese A, et al. Meta-analysis of fecal metagenomes reveals global microbial signatures that are specific for colorectal cancer. Nature medicine. 2019;25(4):679-89.

8. Liss MA, White JR, Goros M, Gelfond J, Leach R, Johnson-Pais T, et al. Metabolic Biosynthesis Pathways Identified from Fecal Microbiome Associated with Prostate Cancer. European urology. 2018;74(5):575-82.

9. Qin J, Li Y, Cai Z, Li S, Zhu J, Zhang F, et al. A metagenome-wide association study of gut microbiota in type 2 diabetes. Nature. 2012;490(7418):55-60.

10. Liu H, Chen X, Hu X, Niu H, Tian R, Wang H, et al. Alterations in the gut microbiome and metabolism with coronary artery disease severity. Microbiome. 2019;7(1):68.
